# Impact of climate change and urban development on the flora of a southern European city: analysis of biodiversity change over a 120-year period

**DOI:** 10.1038/s41598-019-46005-1

**Published:** 2019-07-01

**Authors:** Mirko Salinitro, Alessandro Alessandrini, Alessandro Zappi, Annalisa Tassoni

**Affiliations:** 1 Department of Biological Geological and Environmental Sciences, University of Bologna, Via Irnerio 42, 40126 Bologna, Italy; 2 Institute for Cultural Heritage, Via Galliera 21, 40121 Bologna, Italy; 3 Department of Chemistry “Giacomo Ciamician”, University of Bologna, Via Selmi 2, 40126 Bologna, Italy

**Keywords:** Plant ecology, Climate-change ecology

## Abstract

Ecological studies in cities are not only aimed at investigating floristic diversity, but also represent informative test cases for understanding ecological system dynamics and responses to urban and climate changes since cities represent microcosms of environmental changes happening globally. The city of Bologna was selected as a case study since two specific and complete studies have been carried out in a 120-years timespan, one in 1894 and one in 2018. Since 1894, a large increase occurred in the number of taxa (families from 41 to 101, species from 176 to 477) and alien species (from 22 to 144), with a 65% total species turnover. The comparison of species life forms pointed out a noticeable recent expansion of phanerophytes and geophytes at the expense of therophytes and hemicryptophytes. The correlation between urbanistic features and plant richness indicated that the main factor affecting plant richness is the presence of green spaces (parks, tree lines, flowerbeds, etc.). Analysis of variation in Ellenberg’s indicator values over the last 120 years evidenced a shift toward shade-tolerant species, mainly connected to the increased presence of parks and trees within the city. Climate change and the presence of artificially irrigated areas within the city has led to an increase in both hygrophilous and drought-resistant species. In particular, the temperature index showed a significantly higher amount of macrothermal species in accordance with a warmer climate and the urban heat island effect.

## Introduction

The world’s population is experiencing a dramatic shift from rural to urban living. Whereas in 1900 only 10% of people were residing in cities, this number now exceeds 54% and is expected to increase to 66% by 2050^1^. The development of urban environments is one of the principal causes of land use change worldwide^2^ and believed to be the major cause of biodiversity alteration. Although floristic diversity^3^ in urban areas is generally poorly documented, inside cities where comprehensive surveys have been undertaken plant species biodiversity is often remarkably high^2^. General data indicate that urbanization reduces native species diversity at a regional scale, while, on the contrary, total plant species richness often increases in the city centres in comparison to wildlands, due to the highly heterogeneous patchwork of urban habitats coupled with the human introduction of exotic species^4^. In addition, in some semi-natural environments within cities, endangered native plant species show consistent populations^5,6^ making urban habitats preferential sites for their conservation.

It is argued that with the increase of global travel, which has led to a greater circulation of plant species and the spread from gardens of introduced alien plants^7–10^, there has been an enhancement of species richness counterbalancing or even exceeding the potential local extinction of native species^11,12^. This high number of non-native species within cities may actually improve ecosystem services (e.g. air quality, pollinator insect diversity)^13,14^ and it is expected that the establishment of garden ornamental plants will keep increasing in the future^15^.

Ecological studies in cities are not only aimed at investigating floristic diversity and connected ecosystem features; since cities represent microcosms of environmental changes happening globally, they can serve as informative test cases for understanding ecological system dynamics and responses to climate and land changes^4^. Several previously published historical floristic/ecological studies considered comparisons over long periods of time (Table 1). These studies are of great historical and botanical value as they reflect the situation preceding most anthropogenic interventions, such as industrialization and increasing household density. In fact, original site conditions in urban habitats have changed tremendously and present living conditions for plants are completely different from those occurring during the last century^16^. Past information, climate records and urban structure of the study sites make it possible to evaluate the influence exerted by humans on local floras^17^.

**Table 1:** Papers comparing urban floristic changes over long periods ( > 50 years) of central European and world cities.

Country	City	Covered period	Year number	Reference
Germany	Frankfurt/Main	1800–2000	200	Gregor *et al*.^15^
Czech Rep.	Plzen	1880–1990	110	Chocholouskova & Pyšek^17^
Germany	Leipzig	1867–1989	122	Klotz & Gutte^48^
Switzerland	Zürich	1839–1998	152	Landolt^29^
Holland	Brussels	1940–2001	61	Godefroid^16^
Germany	Halle	1687–2008	321	Knapp *et al*.^36^
Russia	Astrakhan	1882–2003	121	Sal’nikov & Pilipenko^38^
U.K.	Plymouth	1880–1998	118	Kent *et al*.^49^
Czech Rep.	Moravia	1908–2005	97	Lososová & Simonová^50^
Australia	Adelaide	1836–2002	166	Tait *et al*.^11^
Germany	Düsseldorf	1848–1983	135	Klotz^51^

In this context, the city of Bologna can be considered a perfect case study for the assessment of long-term floristic changes since two floristic surveys were carried out with a gap of more than 120 years. The first study dates back to 1894^18^, when the city still had its medieval arrangement, and aimed at describing the species occurring in the city and their habitats, while the second one was carried out in 2018^19^ in the same sampling area in order to create an updated checklist of urban plant diversity. Throughout the paper, we will refer to the historical flora of 1894 as “Gabelli, 1894” and to the modern flora of 2018 as “Salinitro *et al*., 2018”.

Taking into consideration both available datasets, the present report aims at: (i) describing the changes in species richness and composition that occurred over a 120-year time period; (ii) analysing the current presence of alien species compared to the past and their role in overall floristic richness of the city; (iii) evaluating changes in species life forms and their ecological requirements using Ellenberg indicator values (EIVs); (iv) correlating urban flora with climate records and urban changes.

## Methods

### Study area

The city of Bologna is located in the Po valley (Northern Italy), close to the Apennine mountains (latitude of 44°30′27″N; longitude 11°21′05″E), with an average altitude of 54 m a.s.l. (http://www.comune.bologna.it/media/files/f2_relazionegeologica.pdf).

The urban soil below the city of Bologna is an assemblage of river sediments (sand and clay) and anthropic deposits^20^. In the area corresponding to the most ancient part of the city founded by Romans, superficial layers are constituted by anthropic rubble, while clay and anthropic deposits are alternated in later urbanized areas like the medieval part (http://ambiente.regione.emilia-romagna.it). Bologna is crossed by two natural streams and numerous artificial canals deriving water from the adjacent Reno and Savena rivers. This dense water system was buried underground in the early 1900s, resulting in the almost complete absence of water inside the city. At present only a few hundred meters of the Reno canal remain unburied, representing the only residual humid habitat inside the city centre.

### Climatic changes in the last decades

A study conducted in 2013 by ARPAE Emilia-Romagna (http://storage.provincia.re.it) revealed a clear trend of regional climate change that also involved the city of Bologna. The study reported measured temperatures and precipitations in the period 1961–2012, pointing out that since the 1990s an average increase of about 2 °C during the summer period occurred. This rise in temperature led to a failure of the hydro-climatic balance, so that the precipitation-evapotranspiration ratio has been negative since 1985 for most of the years. Since the 1970s, the presence of the urban heat island effect (U.H.I.) in the city of Bologna was also assessed, showing a general increase of 2.7 °C of winter temperatures in the city centre compared to the countryside^21^.

### Urban changes in the study area during the last 120 years

**Figure 1:**
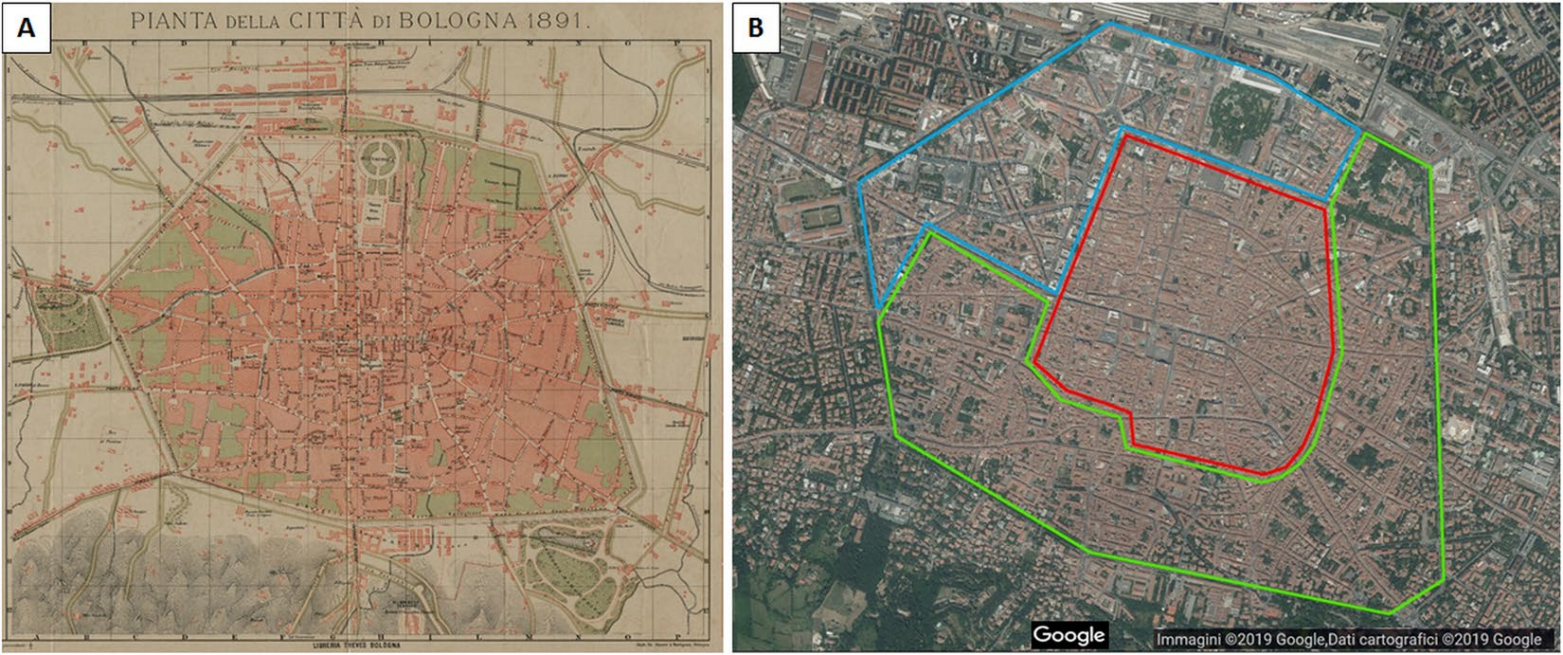
Study area corresponding to the historical centre of the city of Bologna. (**A**) Map of 1891 (from Biblioteca Archiginnasio Bologna, BCABO, GDS, Raccolta piante e vedute della città di Bologna, Cartella 8, n.79, ID 143); (**B**) 2018 satellite image downloaded from Google Maps (www.google.com/maps) and graphically elaborated with the ArcMap programme (version 10.1, ESRI, www.esri.com) by M. Salinitro on the basis of the dataset “Salinitro *et al*. 2018” (Supplementary Table S1). HBA, historical building area (blue); NBA, new building area (green); OCC, old city centre (red).

At the end of the 19th century, Bologna still looked like a medieval city (Fig. 1A) with a complete circle of walls (built around the XIV century), narrow roads mainly made of packed-earth and very low vehicular traffic. Public urban parks were almost completely absent, while large agricultural areas (nowadays completely disappeared) were present within the historical city centre located inside the walls. Bare brick walls were common while roads were mainly unpaved or cobblestone-covered, offering the perfect conditions for plant settling. Natural local materials were used, like wood and gypsum in buildings and pebbles or trachyte slabs in main streets. In this urban context, the first complete study on the urban flora of Bologna was carried out by Lucio Gabelli in 1894^18^. At the beginning of the 20th century, however, the city was completely renovated due to the large industrial expansion and the increase of the urban population. The ancient XIV century walls were almost completely demolished together with the moat and embankment surrounding them. All the main streets of the city were enlarged, rectified and paved, and the agricultural areas within the city centre were replaced by new buildings. Local materials were replaced and at present, streets are covered with asphalt, trachyte plates and porphyry cubes. Gypsum and wood lost importance as building materials, while brick walls were mostly plastered. Following the Second World War, the city experienced a great expansion outside its historical core, eliminating all the rural areas still present close to the city. To counterbalance the lack of green areas, tree lines were planted and small parks were created within the city centre (http://dru.iperbole.bologna.it/normativa/prg-1985; http://www.rapu.it/ricerca/scheda_piano.php?id_piano=138; http://ambiente.regione.emilia-romagna.it).

Nowadays, Bologna’s historical city centre corresponds to the part of the city formerly surrounded by the ancient city walls of the XIV century. In this area, three zones with different building construction types are recognizable (Fig. 1B). The new building area (NBA) was created after the Second World War and consists of tall buildings and wide streets with scarce vegetation mostly confined to public parks. The old city centre (OCC), located in the central part of the city, inherited part of the roman and medieval structures. It consists of narrow streets (except those widened at the beginning of the 1900s) with 3–4-storey buildings and lacks public green areas. Finally, in the southern and eastern part of the city, there is an area with historical buildings (HBA), ranging from XVIII century palaces to modern houses largely endowed with internal courtyards (Fig. 1B). This area, though poor in public parks, is quite green with generally wide streets.

### Data sources

The study area taken into consideration in the present paper corresponds to Bologna’s historical city centre identified as the part of the city formerly surrounded by the walls built in the XIV century. This particularly restricted study area was selected in order to allow the comparison of the data acquired in “Salinitro *et al*.”^19^, that aimed at a comprehensive survey of total plant richness, with those of “Gabelli”^18^, a study meant to describe the species occurring in the city and their habitats. Making a comparison between Bologna’s current flora and the one present over 120 years ago is certainly not an easy task, in particular due to the different methodologies followed for data collection and the changes of plant scientific names that occurred over the years. In fact, in “Salinitro *et al*.”^19^, data were generated from direct observations based on a network of transects that uniformly covered the study area. In this way it was possible to link every observation to a specific locality, allowing for a spatial analysis of the urban flora. The data described in “Gabelli”^18^ were instead a mix of direct observations, done by the author himself, and reports by two other previous authors, without following a scientific sampling methodology. Species localities in the ancient study were rarely recorded (i.e. with the name of a street or an ancient city door), instead most species were described as common in certain habitats (i.e. along the roads, in paved areas, in gardens, etc.) or defined as widespread in the whole city. Plant names cited in the ancient study were often outdated (not used anymore in modern plant systematics) and, even though common names were not reported in “Gabelli”^18^, the linkage between ancient synonym and modern name was always possible.

Finally, data collection based on reports in “Gabelli”^18^ has caused an inevitable bias in the estimation of species abundance. In fact, most of the old reports focused on unusual or rare species in the urban area, leading to an enrichment in rare species and an underestimation or lack of some common species, as in the case of *Plantago lanceolata* L., *Sagina apetala* Ard. and *Solanum nigrum* L., which were not recorded but certainly already present.

Despite the limits listed above, the old floristic study constitutes an important and unique source of data on the urban flora of Bologna at the end of the 19^th^ century, from which it is possible to draw relevant conclusions on the influence that climate change, urban planning and floricultural trends have had over the last 120 years on Bologna’s urban flora biodiversity.

### Data processing

To compare the current flora to that of 1894, and to correlate them with climate and urban changes, Ellenberg indicator values (EIVs)^22^ were applied to the Italian flora^23^ and to exotic species^24^. As EIVs for neophytes were not available in the literature, the indices were calculated by comparison with known species growing in the same habitat following the method of Domina *et al*.^24^. To ascertain the difference between the modern and ancient flora, the Sørensen similarity index was calculated^25^.

A standardization of the two datasets with respect to botanical names and geographical localities was performed. All the historical plant names were linked to modern ones using the PlantList website (www.theplantlist.org) which provided a complete list of synonyms associated with each species. No ambiguities were evidenced in correlating old and current plant names. When possible, the city’s historical plant distribution was inferred by linking the names of the old toponyms to modern ones. Species were considered present in specific ancient habitats (i.e, road sides, gardens, etc.) when they were also detected in the corresponding modern habitat. Plants defined as common or widespread in the 1894 city were also taken into consideration for the whole modern city area. Despite all efforts to reconstruct the spatial distribution of the ancient flora, the lack of detailed information in the “Gabelli, 1894” dataset did not allow a spatial comparison with the “Salinitro *et al*., 2018” dataset.

To determine how species richness in different building areas was influenced by paving materials, and which materials mainly characterized each type of building area, a non-parametric multi-dimensional scaling (NMDS) was performed (software *R*, package Vegan^26^). Manhattan distance was used for computation, convergence was reached after 100 iterations and the stress of the optimal solutions was 0.0397. To analyse the distribution of species according to their presence or absence in different urban building areas, a PCA analysis was performed. The G-test (software *R*, package DescTools^27^) was used to evaluate significant differences between the two datasets with regard to EVIs, life forms, main botanical families and chorotypes. Graphic elaborations were carried out using Microsoft Excel and *R* software (R Core Team, Vienna, Austria).

The whole set of raw data is available in Supplementary Table S1.

## Results

### General floristic trends

**Figure 2:**
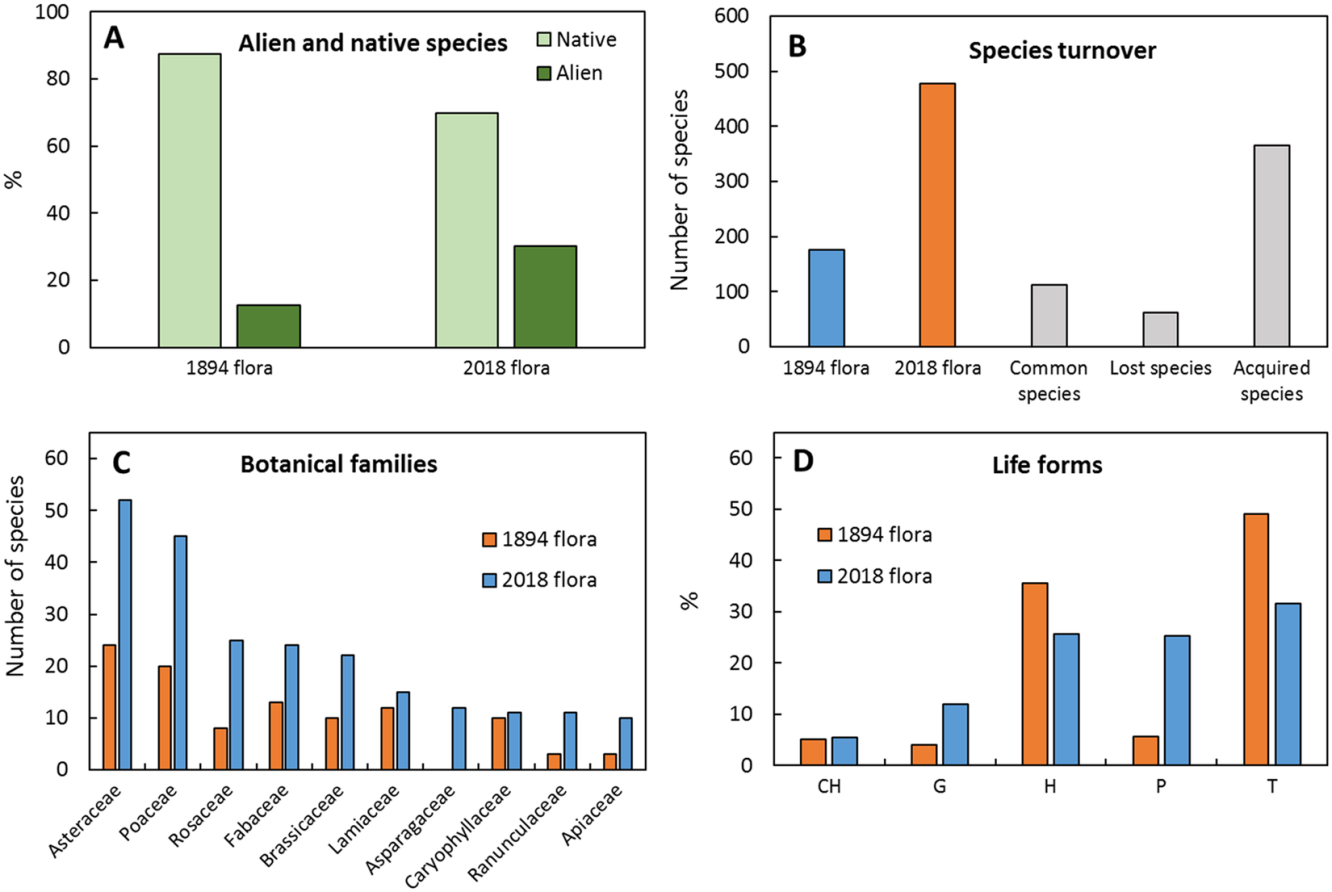
Comparison of floristic data reported in the two reference studies, Gabelli^18^ and Salinitro *et al*.^19^. (**A**) Percentage of alien and native species. (**B**) Total species and number of lost, acquired and common species. (**C**) Most abundant botanical families, the statistical difference in the distribution of families was tested by G-test (*p* < 0.01). (**D**) Life forms: CH = chamaephytes; G = geophytes; H = hemicryptophytes; P = phanerophytes; T = therophytes. The statistical difference in the distribution of families was determined using the G-test (*p* < 0.01).

**Figure 3:**
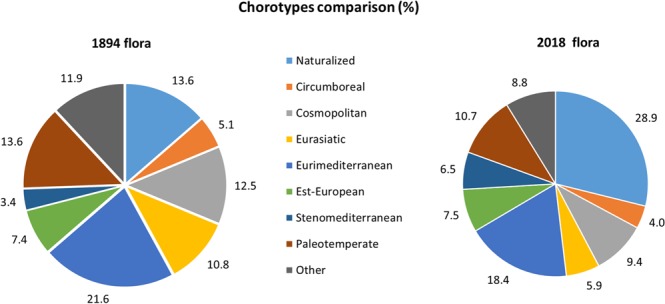
Comparison of chorotypes between “Gabelli, 1894”^18^ and “Salinitro *et al*., 2018”^19^. Some chorotypes have been grouped into a single category, such as Naturalised (Naturalised + Cultivated), Est-European (Eurosiberian + Eurocaucasian), and Cosmopolitan (Cosmopolitan + Subcosmopolitan). The statistical difference in the distribution of chorotypes was tested by G-test (*p* < 0.01).

When comparing floristic data in”Gabelli, 1894” with those in “Salinitro *et al*. 2018”^18,19^, a big increase in the number of recorded taxa could be detected. Recorded botanical families increased from 41 to 101, genera from 138 to 306 and species from 176 to 477 (Fig. 2B). In 120 years, the number of alien species has more than doubled, from less than 12.5% to the current 30.1% (i.e. from 22 to 144 species of the respective total amounts) (Fig. 2A). A large turnover in species was detected, with only 113 species still in common between the two floras; the Sørensen similarity index value was 0.35, indicating a mean 65% of species turnover in 120 years. Over this time period some of the most represented botanical families (Fig. 2C) have changed. Apart from *Asteraceae* and *Poaceae* species that resulted the most abundant in both floras, those of the *Fabaceae* and *Lamiaceae* families decreased. In contrast, the *Asparagaceae* family, not recorded in the old study, entered in the top ten, while the *Rosaceae* family underwent a significant increase of 68%. The species newly introduced in Bologna’s contemporary flora^19^ (mostly alien ornamental) belong to different botanical families, with the result that 30% of the current botanical families are represented by only one species (data not shown). A comparison between the life forms (Fig. 2D) of the ancient and present floras pointed out a noticeable increase in the incidence of phanerophytes (from 6 to 25%) and geophytes (from 4 to 12%). Therophytes and hemicryptophytes decreased (from 49 to 32% and from 36 to 26%, respectively) while no change was found for chamaephytes (around 5%). In addition, the chorological spectra deeply changed between the ancient and the modern floras (Fig. 3), evidencing a doubling of naturalised (from 13.6 to 28.9%) and stenomediterranean species (from the 3.4 to the 6.5%). As a consequence, all the other classes experienced a decrease in comparison with “Gabelli, 1894”, in particular the eurasiatic species (from 10.8 to 5.9%).

### Changes in ecological requirements: Ellenberg indicator values analysis

**Figure 4:**
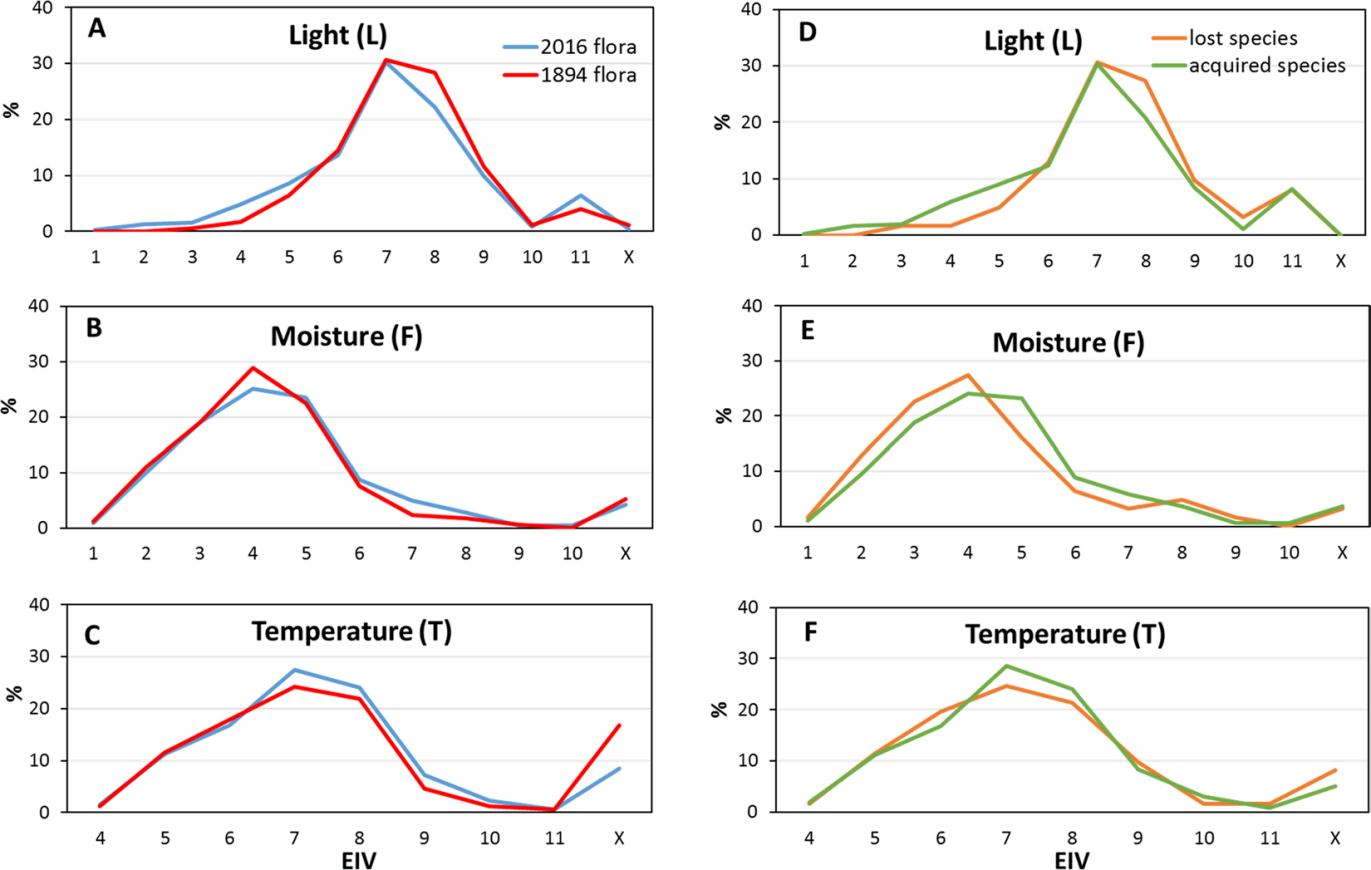
Ellenberg indicator values for “Gabelli, 1894”^18^ and “Salinitro *et al*., 2018”^19^ and for acquired and lost species of the modern and 1894 floras. (**A**,**D**) light (L) index; (**B**,**E**) moisture (**F**) index; (**C**,**F**) temperature (T) index. The statistical difference was tested by G-test (*p* < 0.05).

When comparing Ellenberg indicator values (EIVs), associated to the two datasets several interesting differences were revealed. The comparison was performed by taking into consideration light (L), moisture (F), temperature (T), reaction (R), continentality (K) and nutrients (N) EIVs, while the salinity (S) index was not included in the analysis since most of the species recorded in both studies were from non-saline soils. Differences between the “Gabelli, 1894” and “Salinitro *et al*., 2018” floras were detected analysing the two whole datasets and lost and acquired species of the modern flora with respect to the old one were highlighted (Fig. 4).

K, R, N indexes did not show significant changes (*p* = 0.456, *p* = 0.345, *p* = 0.746 respectively) between the two datasets and, therefore, they were not included in Fig. 4. The complete set of data is available in Supplementary Table S1. The light (L) index pattern of the two whole floristic datasets (Fig. 4A) indicates that shade-tolerant and intermediate species (categories 1–5) experienced an increase between 1 to 3%, moderate heliophilous species (categories 8 and 9) showed a strong decrease (respectively, 6 and 2%) while highly heliophilous species (category 11) experienced an increase of 2.4% (*p* = 0.053). These general changes were even more emphasized when lost and acquired species (of the modern flora) were compared, with exception of category 11 (*p* = 0.032) (Fig. 4D). The moisture (F) index (Fig. 4B) showed no significant changes in drought-resistant species (categories 1 to 3), while a 3.7% decrease was observed in category 4. Intermediate categories (6–8) increased by between 4.7 and 3.5%, while no significant changes were observed for hygrophilous species (categories 9–10) and moisture adaptable species (category X). Overall, the changes in F index were associated with a *p* value of 0.081. On the other end, when the moisture indexes of lost and acquired species of the present flora were compared (Fig. 4F), a 4% decrease of drought-resistant species (categories 2–4), and an enrichment (2.7 to 7%) of intermediate tolerant species (categories 5 to 7) were found (*p* = 0.029). The temperature (T) index in both studies starts from category 4 as no plants tolerant to very low temperatures (e.g. alpine or sub-alpine species) were or are actually present in the Bologna study area. The analysis of T indexes (Fig. 4C) indicates that microthermal and mesothermal species (categories 4 to 6) did not change in 120 years. On the contrary, macrothermal species increased by 3% (categories 7 to 10) at the expense of plants adapted to a wide range of temperature. In fact, the number of species belonging to category X halved in 120 years (from 16.8 to 8.5%) (*p* = 0.033). A similar pattern was found when comparing lost and acquired species (Fig. 4E) (p = 0.024).

### Species diversity in relation to urban structure

During the present study, particular attention was given to differences in the city structure and the distribution of different building materials. Building materials (especially paving materials) are in fact primary selective factors for plant colonisation.

**Figure 5:**
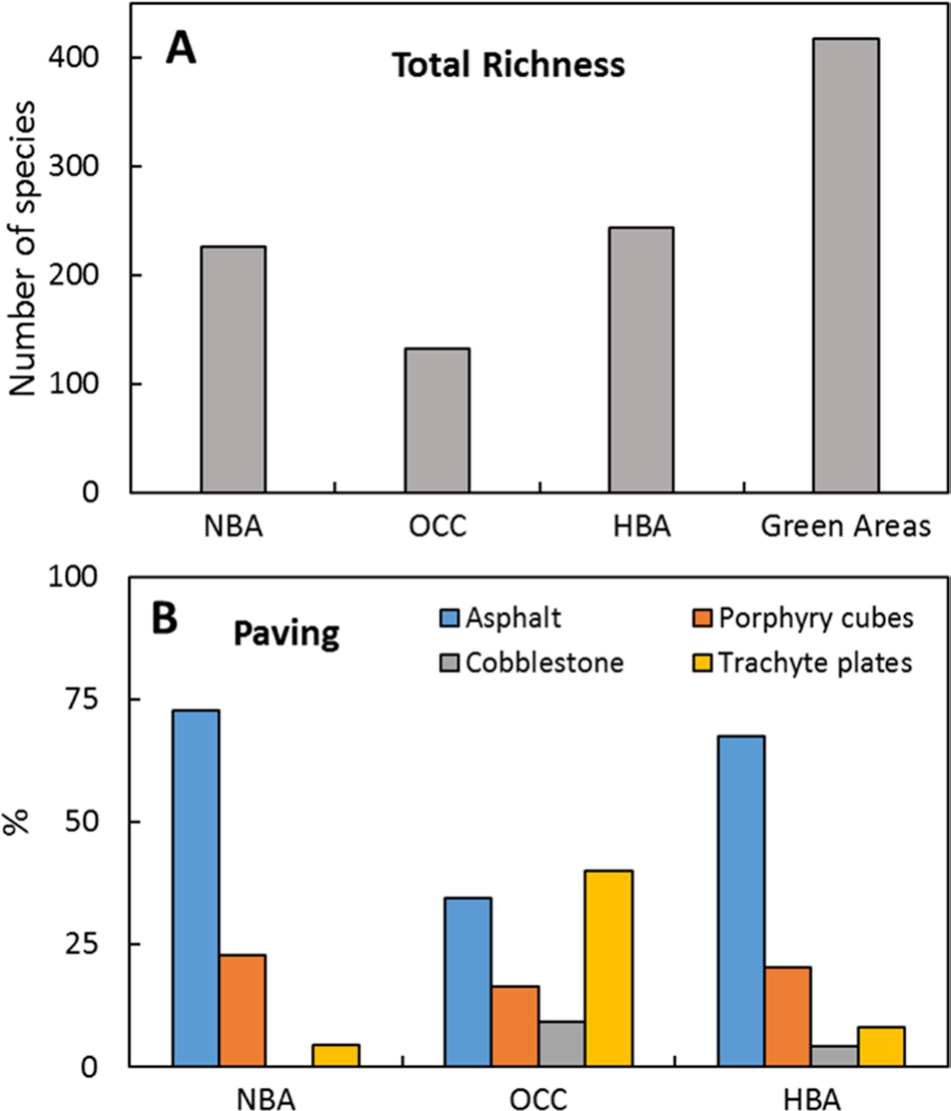
Floristic richness variation in the different building areas. (**A**) Total species richness in different city centre areas. HBA, historical building area; NBA, new building area; OCC, old city centre. (**B**) Type of paving coverage in the different urban areas.

**Figure 6:**
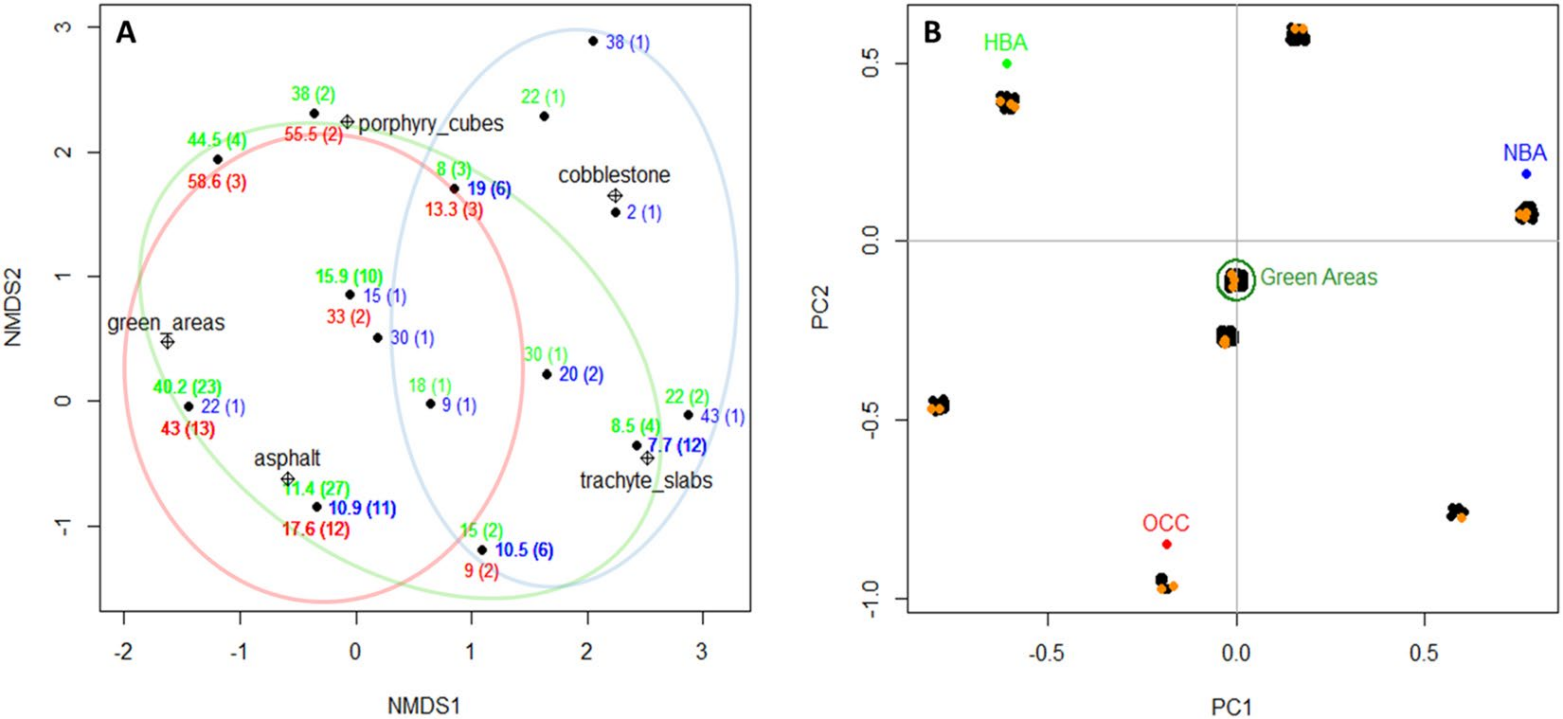
Species richness in Bologna’s different urban areas and correlation with paving materials. (**A**) NMDS analysis showing the distribution of transects (black dots) according to the presence of different paving materials (empty crossed-squares). Black dots represent several overlapped transects with similar features. Numbers represent the average richness and (in brackets) the amount of transects. Blu colour, HBA; green, NBA; red, OCC. (**B**) PCA analysis showing the grouping of species according to their presence in the different building areas. Several species are overlapped in each group of black dots, each dot represents a single species. Dark green circle: species growing exclusively in green spaces. Orange dots represent typical species of each habitat (see text for details). PC2 vs PC3 was used to allow better species grouping. In all the figures, HBA: historical building area, NBA: new building area, OCC: old city centre.

Large part of the current floral richness (477 plant species) of Bologna’s historical city centre^19^ is ascribable to green areas, which alone contain 418 species (Fig. 5A). Among the three different building areas identified (Fig. 1B), new building area (NBA), old city centre (OCC), and historical building area (HBA), several differences were observed regarding the presence of green spaces and the type of flooring used that affect floristic richness and composition. The presence of green spaces adjacent to streets, such as gardens, flowerbeds or tree-lines, was quite different: 40.6% in NBA, 36.2% in HBA and only 12.7% in OCC. The most widespread paving for HBA and NBA was asphalt, while trachyte plates were more abundant in OCC. The presence of porphyry cubes was almost constant among the three building areas, while cobblestone paving was scarcely represented (Fig. 5B). The influence of paving materials on total species richness was shown in Fig. 6A. Transects richest in species were primarily characterized by the presence of green spaces and asphalt, while, on the contrary, those showing porphyry cubes and trachyte slabs were the poorest. Accordingly, decreasing values of species richness were detected for HBA, NBA and OCC containing on the whole 244, 226 and 132 species, respectively (Fig. 5A). Differences in structure and materials present in each type of building area not only affected species richness but also led to the differentiation into three different sub-floras (Fig. 6B). Eight species were exclusive to OCC, among which roof plants, like *Polypodium interjectum* Shivas and *Sedum dasyphyllum* L.; 58 species were only found in HBA, among which many ornamental garden plants, such as *Alcea rosea* L., *Buxus sempervirens* L. and *Elaeagnus pungens* Thunb.; 47 species grew exclusively in NBA, among which some exotic species of sunny places, like *Dysphania pumilio* (R. Br.) Mosyakin & Clemants, *Delosperma cooperi* (Hook.f.) L. Bolus and *Lepidium virginicum* L.; and 159 species were only detected in green areas, among them many rare native species of shady habitats, like *Scilla bifolia* L., *Silene flos-cuculi* L. and *Vinca minor* L. A large number of species (39.7%) were shared between two or more building areas, such as *Mahonia japonica* (Thumb.) DC., *Papaver rhoeas* L. in NBA and HBA and *Ailanthus altissima* (Mill.) Swingle, *Plantago major* L., and *Stellaria media* L. (Vill.) present in all three building areas.

## Discussion

Comparison between the 1894 and 2018 floristic data^18,19^ clearly demonstrated the impact of climatic and urban changes on floristic diversity and assemblage in Bologna’s historical city centre.

In agreement with several published reports on other cities^11,28,29^ worldwide, the present results point out that the total number of species increased dramatically over a 120 years timespan, in particular, the number of alien species which doubled (from 12.5 to 30.1% of total species) (Fig. 2A). The species number increase seems to be a common trend in many urban areas^17,28,30–32^, nonetheless the large number of species detected in “Salinitro *et al*. 2018” could also be partly due to an underestimation of total species richness in “Gabelli. 1894”. However, the recorded amount of alien species was in line with European and world averages, e.g. 25.2%^28^ and 28%^12^. A similar increase was also found when higher taxa levels (genera and families) were analysed and, in general, the high diversity in cities could be ascribed to the large variety of urban microhabitats and, above all, to the human voluntary and non-voluntary introduction of species for ornamental purposes^7,8,33,34^. In fact, the cultivation of ornamental plants for leisure and decoration represents the primary factor contributing to the increase of neophytes^9,15^. Asteraceae and Poaceae were found to be the most widespread botanical families in Bologna’s current flora, similarly to many other urban areas in the world^35^. The increase of *Asparagaceae*, *Rosaceae* and *Oleaceae* could be mainly ascribed to the naturalisation of cultivated species. Interestingly, 65% of recently lost species are represented by indigenous ones, as previously detected also in other cities such as Zürich^29^ (about 60%). As a confirmation of previous considerations, the Sørensen similarity index, indicating the species turnover and calculated between Bologna’s ancient and modern flora, is significantly lower (0.35) compared to other cities like Zürich, Brussels and Plzen^16,17,29^ (on average 0.80). This large species turnover detected in Bologna over 120 years may reflect the urbanistic changes the city centre underwent from the beginning of the 20^th^ century, like the disappearance of cultivated fields and medieval walls, which dramatically reduced the presence of green areas in the city^36^. Therefore, the huge floristic turnover connected to urban modification regarded in particular the disappearance of crop species^36^. “Gabelli, 1894”^18^ reported fruit-bearing trees, cereals and common weeds as widely naturalised inside the city centre. In Bologna’s current flora, field species, like *Legousia speculum-veneris* (L.) Chaix, *Echium vulgare* L. and *Matricaria chamomilla* L., and cultivated crops like *Zea mays* L., are no longer present in the urban area due to the intense urbanization process that occurred over the last century and changed the shape of the city (Fig. 1). A similar reduction in field species has previously been observed in other cities like, e.g., Brussels^16^. On the other hand, because of the choice of a restricted and highly urbanized study area for both reference studies,^18,19^ stochastic processes connected to extreme anthropogenic influences are predominant in urban environments^37^.

When taking into consideration life form variations, the current increase in phanerophytes (Fig. 3) seems to be a common trait connected with the increase of naturalised species in urban floras^17,38^. Similarly, the growing amount of woody species (mainly ornamental plants) has been hypothesized to be in relation with the naturalisation of cultivated species^7,8,17,38^. Due to the strong increase of woody plants, the total percentage of therophytes and hemicryptophytes decreased (Fig. 3). Nonetheless, the current flora of Bologna is still dominated by therophytes and hemicryptophytes, while most of the newly introduced phanerophytes occur only temporarily, without forming persisting populations, as was also pointed out by Chocholousková *et al*.^17^ with regard to the flora of the city of Plzen. The therophytes/hemicryptophytes ratio also decreased in the period from 1894 (1.36) to 2018 (1.23) indirectly confirming climatic data that evidence an increase of summer drought over the years (http://storage.provincia.re.it) which generally favoured the growth of annual plants. Moreover, the increase of geophytes species (Fig. 3) (from 4 to 12%) could be explained by the recent naturalisation of some ornamental species (e.g. *Iris germanica* L.), even if an underestimation of this type of plants in the past study could be hypothesized, as some common certainly present native species, like *Allium vineale* L., were not recorded. Similar results were reported for several other European cities^39,40^. However, the most glaring sign of the adaptation of the current flora to a warmer and drier climate is the increase in stenomediterranean species with respect to the past century. In fact, as pointed out by Hruška^41^ and Interdonato *et al*.^42^, a high ratio of stenomediterranean/eurimediterranean species is typical of cities in southern Italy, which are usually on average much warmer than the city of Bologna located in northern Italy. Therefore, these cities may have started to host plants coming from warmer world regions^37^.

The comparison of EIVs (Fig. 4) pointed out the ongoing adaptation of the flora to the changes that occurred in the city over the last century. In particular, an expansion of all shade-tolerant categories at the expense of heliophilous ones was observed (Fig. 4A,D). Analogous findings were reported for the cities of Plzen, Brussels, Zurich and Halle using data collected over a similar timespan^16,17,29,36^. The observed changes could be correlated to urbanistic and structural modifications of the city, which include a general enlargement of the main avenues and planting of trees in parks and streets^36^. The currently present tree lines can, therefore, be considered responsible for the settling of many understory and sciophilous species^29,36^, like *Hedera helix* L. and *Trachycarpus fortunei* (Hook.) H. Wendl. Pteridophytes also largely contributed to the shade-tolerant plant category (light index categories 1 to 5) while they were poorly represented in the historical flora list, being probably underestimated. Moisture index changes (Fig. 4B,E) mainly reflected the anthropic changes of the city, like the widespread use of irrigation systems inside Bologna’s historical centre. This practice inevitably overshadows the effects of climate changes on floral biodiversity, as long as the soil moisture content is artificially modified. F index data collected for Bologna’s city centre showed an increased presence of hygrophilous plants (categories 6 to 8, Fig. 4B) analogously to what reported for several other European cities^43^ like Plzen, Halle and Brussels^16,17,36^. This trend was confirmed by EIVs analysed for lost and acquired species (Fig. 4E) with a net gain of hygrophilous species in the 2018 flora. As previously demonstrated for other cities, for example Boston^44^, irrigated areas turn out to be a shelter for species that otherwise would be no longer present in the area, such as hygrophilous species like *Epilobium montanum* L., *Equisetum arvense* L. and *Eclipta prostrata* (L.) L., which were recently found in Bologna. However, it is not possible to exclude the presence of such species from the 1894 flora. In fact, at that time the city centre was characterized by a wide extension of channels that probably hosted a large number of plants, including many hygrophilous species, which were not recorded by Gabelli as these channels were not investigated.

Analysing the temperature EIV (Fig. 4C,F), an increase in mesothermal and macrothermal (categories 7 to 10) species and a concomitant decrease of tolerant species (category X), was evidenced. A similar trend has been found in most of the cities previously investigated, in agreement with a global temperature increase (more evident in urban environments) in the last decades^16,29,40^. Key players of these biodiversity changes are many Mediterranean (e.g. *Pistacia therebinthus* L. and *Cupressus sempervirens* L.) and alien thermophilic species (e.g. *D*. *cooperi* (Hook.f.) L.Bolus and *Chlorophytum comosum* (Thunb.) Jacques), which in recent years have started to survive the winter given the milder temperatures. The tropicalization of the flora is, in fact, supported by the U.H.I.’s in many European cities^33,45^ which, in particular in the Bologna city area, resulted in an increase of winter low temperatures of 2.7 °C compared to the past^21^. Moreover, the strong decrease of species with a wider tolerance (category X) was confirmed by a shift toward extreme temperatures, especially in summer. In the present data, the maximum plant species richness was detected in green areas (e.g. parks, flowerbeds or tree lines) (Figs 5A and 6A), as confirmed also by similar reports for the cities of Rome^46^ and Zürich^29^ with in the latter case the richest area sampled being the one containing the botanical garden.

The positive correlation between species richness and green areas was further confirmed by the progressive impoverishment of urban biodiversity going from HBA to NBA to OCC, in accordance with the decreasing presence of green areas. Similar patterns were reported for the city of Rome^46^, where the historical centre (approximately corresponding to Bologna’s OCC) and new 1950–1980 building developments (similar to NBA) showed the poorest levels of plant diversity. Our findings also support the thesis that paving materials differently contributed to enhance or decrease street floristic richness (Fig. 6A), suggesting that building materials could have played an important role in characterizing current urban flora. Furthermore, previous results on Bologna^19^ showed a negative correlation between floristic diversity and street width (generally higher in NBA and HBA with respect to OCC) suggesting that the larger modern streets have played an important role in increasing plant richness coupled with the creation of new green spaces.

## Conclusions

The analysed data demonstrated that over 120 years the floristic diversity of Bologna’s historical city centre has deeply changed as a result of modifications in urban architecture, warmer climate conditions and the increasing anthropogenic pressure on the urban environment. In general, climate and urban changes did not lead to a loss of floristic diversity, but rather to a loss of “typicality”, with an almost tripled amount of alien species (mostly introduced ornamental plants^7,8^) and the disappearance of native and crop species, resulting in a homogenisation of the flora^5,47^. Nevertheless, the addition of tree lines along the main streets, the creation of small parks and the increase of irrigated areas around the historical city centre have made the urban landscape more heterogeneous than in the past, creating opportunities for the growth of a wider range of species. This factor coupled with the massive introduction of alien species are believed to be the cause of the general increase of biodiversity over the past 120 years.

## Supplementary information

Supplementary Dataset

## Data Availability

All data generated or analyzed during this study are included in the published article and in Supplementary Table S1.
