# Supplementary material for: Impact of climate change and urban development on the flora of a southern European city: analysis of biodiversity change over a 120-year period
Source: Sci Rep. 2019 Jul 1;9:9464. doi: 10.1038/s41598-019-46005-1 (PMC6603027; doi:10.1038/s41598-019-46005-1)
Supplement: Supplementary file 1 — Supplementary Dataset [file 41598_2019_46005_MOESM1_ESM.pdf]

Manuscript Title: Impact of climate change and urban development on the flora of a southern European city: analysis of biodiversity change over a 120-year period

Authors: Mirko Salinitro, Alessandro Alessandrini, Alessandro Zappi, Annalisa Tassoni

Raw datasets

DATASET:"Salinitro et al 2018"

Species	year	life_form	chorotype	L	T	C	U	R	N	green_areas	OCC	HBA	NBA
Acalypha_australis	2018	T SCAP	AVV NATURALIZZ	8	8	5	4	6	9	x			
Acanthus_mollis	2018	H SCAP	W-STENOMEDIT	7	8	4	3	5	4	x	x	x	
Acer_campestre	2018	P SCAP	EUROP-CAUCAS	5	7	4	5	7	6	x			x
Acer_negundo	2018	P SCAP	AVV NATURALIZZ	8	7	5	5	5	5	x		x	x
Acer_pseudoplatanus	2018	P SCAP	EUROP-CAUCAS	4	7	4	6	6	7	x		x	x
Acer_saccharinum	2018	P CAESP	COLTIV	8	7	5	5	5	5	x			
Achillea_roseoalba	2018	H SCAP	CENTRO-EUROP	7	6	7	3	6	3	x			x
Adiantum_capillus-veneris	2018	G RHIZ	PANTROP	1	8	3	9	5	3	x			
Aegopodium_podagraria	2018	G RHIZ	EUROSIB	5	7	4	6	7	8	x			
Aesculus_hippocastanum	2018	P SCAP	AVV NATURALIZZ	7	7	7	4	5	5	x		x	
Ailanthus_altissima	2018	P SCAP	AVV NATURALIZZ	6	7	5	5	5	5	x	x	x	x
Ajuga_reptans	2018	CH REPT	EUROP-CAUCAS	6	7	4	6	6	6	x			
Albizia_julibrissin	2018	P SCAP	COLTIV	10	9	5	5	6	4			x	
Alcea_rosea	2018	H SCAP	COLTIV	9	8	5	3	6	4	x		x	
Alliaria_petiolata	2018	H SCAP	PALEOTEMP	5	6	5	5	7	9	x			
Allium_ampeloprasum	2018	G BULB	EURIMEDIT	7	7	5	3	6	5	x			
Allium_roseum	2018	G BULB	STENOMEDIT	8	8	4	3	6	5	x			
Allium_triquetrum	2018	G BULB	W-STENOMEDIT	6	9	4	4	4	7	x			
Allium_vineale	2018	G BULB	EURIMEDIT	8	7	5	4	6	7	x		x	
Alopecurus_myosuroides	2018	T SCAP	SUBCOSMOP	6	6	5	6	7	7	x	x	x	x
Amaranthus_blitoides	2018	T SCAP	AVV NATURALIZZ	9	7	7	3	6	9		x	x	x
Amaranthus_blitum	2018	T SCAP	EURIMEDIT	8	8	5	4	6	8	x			x
Amaranthus_deflexus	2018	T SCAP	AVV NATURALIZZ	8	8	5	4	6	9	x	x	x	x
Amaranthus_retroflexus	2018	T SCAP	AVV NATURALIZZ	9	9	7	4	6	9	x		x	x
Amaranthus_tuberculatus	2018	T SCAP	AVV NATURALIZZ	8	8	5	4	6	8			x	
Ammi_majus	2018	T SCAP	EURIMEDIT	11	9	5	4	5	2	x			
Anagallis_arvensis	2018	T REPT	EURIMEDIT	6	6	5	5	6	6	x		x	x

Anisantha_diandra	2018	T SCAP	EURIMEDIT	8	8	5	3	5	4	x			
Anisantha_rubens	2018	T SCAP	S-STENOMEDIT	8	11	5	2	6	2	x		x	
Anisantha_sterilis	2018	T SCAP	EURIMEDIT	7	7	5	4	6	5	x	x	x	x
Anthoxanthum_odoratum	2018	H CAESP	EURASIAT	7	7	5	4	5	3	x			
Anthriscus_sylvestris	2018	H SCAP	PALEOTEMP	7	7	5	5	6	8	x	x		x
Antirrhinum_majus	2018	CH FRUT	W-STENOMEDIT	11	8	4	2	6	1	x	x		x
Apium_graveolens	2018	H SCAP	PALEOTEMP	7	7	5	7	5	7	x			
Aquilegia_sp. (hybrid)	2018	H SCAP	PALEOTEMP	6	6	5	4	7	4	x		x	x
Arabidopsis_thaliana	2018	T SCAP	PALEOTEMP	6	7	5	4	5	4	x	x	x	x
Arctium_minus	2018	H BIENNE	EURIMEDIT	11	5	5	5	8	9	x	x	x	x
Arenaria_serpyllifolia	2018	T SCAP	PALEOTEMP	9	9	5	2	3	1	x		x	x
Arenaria_serpyllifolia_ssp.	2018	T SCAP	SUBCOSMOP	9	5	5	4	6	5	x	x	x	x
Artemisia_verlotiorum	2018	H SCAP	AVV NATURALIZZ	7	7	6	7	5	5	x		x	
Artemisia_vulgaris	2018	H SCAP	CIRCUMBOR	9	7	8	4	6	5	x			x
Arum_italicum	2018	G RHIZ	STENOMEDIT	6	8	4	4	5	5	x		x	x
Arundo_donax	2018	G RHIZ	SUBCOSMOP	8	9	5	5	5	6	x			
Asparagus_acutifolius	2018	NP	STENOMEDIT	6	9	4	2	5	5	x		x	
Asparagus_aethiopicus	2018	G RHIZ	COLTIV	8	9	0	3	2	4	x			
Aspidistra_elatior	2018	G RHIZ	COLTIV	3	8	4	5	3	5	x			
Asplenium_adiantum-nigrum	2018	H ROS	PALEOTEMP	6	7	2	4	2	3		x		
Asplenium_ceterach	2018	H ROS	EURASIAT	9	7	5	2	7	3		x	x	
Asplenium_scolopendrium	2018	H ROS	CIRCUMBOR	2	6	4	5	8	6	x		x	
Asplenium_trichomanes	2018	H ROS	COSMOPOL-TEMP	5	7	5	5	6	4		x	x	
Atriplex_patula	2018	T SCAP	CIRCUMBOR	6	5	5	5	7	5	x			
Avena_barbata	2018	T SCAP	EURIMEDIT	8	8	5	3	7	2	x		x	x
Avena_sterilis	2018	T SCAP	EURIMEDIT	8	9	5	3	6	4	x			
Ballota_nigra	2018	H SCAP	EURIMEDIT	8	6	5	5	6	8	x			x
Bellis_perennis	2018	H ROS	EUROP-CAUCAS	9	5	4	4	6	5	x	x	x	x
Beta_vulgaris	2018	H SCAP	COLTIV	11	7	5	6	6	5				x
Brachypodium_sylvaticum	2018	H CAESP	PALEOTEMP	4	5	5	5	6	6	x			
Bromus_hordeaceus	2018	T SCAP	SUBCOSMOP	7	6	5	4	6	5	x		x	x
Broussonetia_papyrifera	2018	P CAESP	AVV NATURALIZZ	8	7	5	5	5	5	x	x	x	x
Buddleja_davidii	2018	P CAESP	AVV NATURALIZZ	7	5	7	7	4	2	x			

Buglossoides_purpurocaerulea	2018	H SCAP	PONTICA	5	7	6	4	8	4	x			
Buxus_sempervirens	2018	NP	EURIMEDIT	5	8	5	4	8	4	x		x	
Calendula_officinalis	2018	T SCAP	EURIMEDIT	8	7	5	4	5	4	x			
Calepina_irregularis	2018	T SCAP	EURIMEDIT-TURAN	8	8	4	3	5	3	x		x	x
Calystegia_sepium	2018	H SCAND	PALEOTEMP	8	6	5	6	7	9	x		x	x
Campsis_radicans	2018	P LIAN	AVV NATURALIZZ	9	7	5	5	5	4	x		x	
Cannabis_indica	2018	T SCAP	AVV NATURALIZZ	8	7	5	5	5	5			x	
Capparis_spinosa	2018	NP	EURASIAT	9	10	5	2	5	1	x			
Capsella_bursa-pastoris	2018	H BIENNE	COSMOPOL	7	7	5	5	5	4	x	x	x	x
Capsella_rubella	2018	T SCAP	EURIMEDIT	8	9	5	2	4	2	x	x	x	x
Capsicum_annuum	2018	T SCAP	AVV NATURALIZZ	7	7	5	5	5	7	x		x	
Cardamine_hirsuta	2018	T SCAP	COSMOPOL	7	8	5	3	5	4	x	x	x	x
Cardaria_draba	2018	G RHIZ	EURIMEDIT-TURAN	8	7	7	3	8	4	x			
Carduus_pycnocephalus	2018	H BIENNE	EURIMEDIT-TURAN	7	8	4	3	6	3	x		x	
Carex_divulsa	2018	H CAESP	EURIMEDIT	7	6	5	4	5	5	x	x	x	x
Carex_pendula	2018	H CAESP	EURASIAT	5	5	5	8	6	5	x			
Carpinus_betulus	2018	P SCAP	EUROP-CAUCAS	4	6	4	4	6	5	x			
Catapodium_rigidum	2018	T SCAP	EURIMEDIT	8	8	5	2	5	4	x	x	x	x
Celtis_australis	2018	P SCAP	EURIMEDIT	7	8	5	3	7	4	x		x	x
Celtis_occidentalis	2018	P SCAP	COLTIV	7	8	5	3	7	4	x		x	
Centaurea_nigrescens	2018	H SCAP	EUROP	7	6	5	4	5	4	x			x
Centranthus_ruber	2018	CH SUFFR	STENOMEDIT	6	8	4	2	6	1	x			
Cephalanthera_damasonium	2018	G RHIZ	EURIMEDIT	2	5	4	4	7	4			x	
Cephalotaxus harringtonii	2018	P SCAP	COLTIV							x			
Cerastium_brachypetalum	2018	T SCAP	EURIMEDIT	11	7	5	3	7	2	x		x	x
Cerastium_glomeratum	2018	T SCAP	EURIMEDIT	7	7	5	5	5	5	x	x	x	x
Ceratochloa_cathartica	2018	H BIENNE	AVV NATURALIZZ	8	7	5	5	5	5			x	
Cercis_siliquastrum	2018	P SCAP	S-EUROP-SUDSIB	8	7	6	4	7	4	x		x	x
Chelidonium_majus	2018	H SCAP	EURASIAT	6	6	5	5	6	8	x	x	x	x
Chenopodium_album	2018	T SCAP	SUBCOSMOPOL	7	7	5	4	5	7	x	x	x	x
Chenopodium_polyspermum	2018	T SCAP	PALEOTEMP	6	5	5	6	4	8	x			
Chlorophytum_comosum	2018	H SCAP	COLTIV	7	9	4	4	5	4	x		x	x
Chimonanthus praecox	2018	P SCAP	COLTIV							x			

Chondrilla_juncea	2018	H SCAP	S-EUROP-SUDSIB	8	7	5	3	8	5	x	x	x	
Cichorium_intybus	2018	H SCAP	PALEOTEMP	9	6	5	3	8	5	x			x
Cirsium_arvense	2018	G RAD	EURASIAT	8	7	5	4	6	7	x	x	x	x
Cirsium_vulgare	2018	H BIENNE	PALEOTEMP	8	5	5	5	6	8	x	x	x	x
Claytonia_perfoliata	2018	T SCAP	AVV NATURALIZZ	6	6	4	5	7	7	x			
Clematis_vitalba	2018	P LIAN	EUROP-CAUCAS	7	7	4	5	7	7	x	x	x	x
Clerodendrum_trichotomum	2018	P SCAP	COLTIV	8	9	5	6	5	3			x	
Clinopodium_nepeta	2018	H SCAP	OROF S-EUROP	5	7	5	3	9	3	x		x	x
Commelina_communis	2018	G BULB	AVV NATURALIZZ	7	6	5	8	6	2	x	x	x	x
Conium_maculatum	2018	H SCAP	PALEOTEMP	7	8	5	4	5	7	x			
Convolvulus_arvensis	2018	G RHIZ	PALEOTEMP	7	7	5	4	5	5	x		x	x
Cornus_sanguinea	2018	P CAESP	EURASIAT	7	5	5	7	8	5	x			
Corylus_avellana	2018	P CAESP	EUROP-CAUCAS	6	5	4	5	5	8	x		x	
Cotoneaster hissaricus	2018	P CAESP	COLTIV							x			
Cotoneaster_horizontalis	2018	NP	COLTIV	5	8	4	3	5	3	x			x
Crambe_hispanica	2018	T SCAP	EURIMEDIT-TURAN	11	10	4	2	7	3	x			
Crataegus_monogyna	2018	P CAESP	PALEOTEMP	6	7	5	4	6	3	x			
Crepis_capillaris	2018	T SCAP	CENTRO-EUROP	7	6	5	4	5	3	x		x	
Crepis_foetida	2018	T SCAP	EURIMEDIT	11	9	5	2	6	2	x		x	x
Crepis_pulchra	2018	T SCAP	EURIMEDIT	8	6	5	4	5	5	x			x
Crepis_rhoeadifolia	2018	T SCAP	SE-EUROP	7	6	6	4	6	3	x			
Crepis_sancta	2018	T SCAP	EURIMEDIT-TURAN	11	9	6	2	6	2	x	x	x	x
Crepis_vesicaria	2018	H BIENNE	EURIMEDIT	8	8	3	3	6	2	x		x	x
Cupressus_sempervirens	2018	P SCAP	E-EURIMEDIT	7	7	6	3	6	3	x			
Cuscuta_campestris	2018	T PAR	AVV NATURALIZZ	8	7	5	4	6	5	x			
Cyclamen_hederifolium	2018	G BULB	N-STENOMEDIT	4	8	5	5	5	5	x			
Cymbalaria_muralis	2018	T SCAP	N-EURIMEDIT	7	7	5	2	5	3	x	x	x	x
Cynodon_dactylon	2018	G RHIZ	COSMOPOL	8	8	5	4	6	4	x	x	x	x
Cyperus_eragrostis	2018	T SCAP	PALEOTEMP	8	9	5	10	6	6				x
Cyrtomium_falcatum	2018	H ROS	COLTIV	2	7	5	5	5	6	x			
Cystopteris_fragilis	2018	H CAESP	COSMOP	5	4	4	5	1	1		x	x	
Cytisus_sp.	2018	NP	COLTIV	6	6	5	4	6	0			x	
Dactylis_glomerata	2018	H CAESP	PALEOTEMP	7	6	5	4	5	6	x			x

Danae_racemosa	2018	G RHIZ	COLTIV	3	4	5	4	4	6	x			
Daucus_carota	2018	H BIENNE	PALEOTEMP	8	6	5	4	5	4	x			
Delosperma_cooperi	2018	CH SUCC	COLTIV	9	9	5	1	6	1	x			x
Dichondra_micrantha	2018	G RHIZ	AVV NATURALIZZ	5	8	5	6	3	2	x	x	x	x
Digitaria_ischaemum	2018	T SCAP	SUBCOSMOP	7	6	5	5	2	3	x		x	x
Digitaria_sanguinalis	2018	T SCAP	COSMOPOL	7	7	5	3	6	4	x	x	x	x
Diospyros_lotus	2018	P SCAP	COLTIV	9	7	5	5	5	4	x		x	
Diplotaxis_muralis	2018	T SCAP	EURIMEDIT-SUBATL	8	8	3	3	5	5	x			
Diplotaxis_tenuifolia	2018	H SCAP	SUBATLANT	8	7	5	4	6	5	x		x	x
Dipsacus_fullonum	2018	H BIENNE	EURIMEDIT	6	8	5	7	5	5	x			
Dittrichia_graveolens	2018	T SCAP	EURIMEDIT-TURAN	11	8	6	3	7	7				x
Dittrichia_viscosa	2018	H SCAP	EURIMEDIT	11	8	5	3	7	9	x		x	x
Dryopteris_filix-mas	2018	G RHIZ	SUBCOSMOP	3	7	5	5	5	6			x	x
Dysphania_pumilio	2018	T SCAP	AVV NATURALIZZ	8	7	5	2	5	5				x
Echinochloa_crusgalli	2018	T SCAP	SUBCOSMOP	6	7	5	7	6	8	x	x	x	x
Eclipta_prostrata	2018	T SCAP	NEOTROPIC	7	7	5	7	5	7	x		x	x
Elaeagnus_angustifolia	2018	P SCAP	AVV NATURALIZZ	9	7	5	3	6	2	x		x	
Elaeagnus_pungens	2018	P CAESP	COLTIV	5	8	4	3	5	5	x		x	
Eleusine_indica	2018	T SCAP	COSMOP	11	8	5	2	7	2	x	x	x	x
Elymus_repens	2018	G RHIZ	CIRCUMBOR	7	7	7	5	6	8	x		x	x
Epilobium_hirsutum	2018	H SCAP	PALEOTEMP	7	8	5	7	6	6	x			
Epilobium_montanum	2018	H SCAP	EURASIAT	6	4	5	5	4	4				x
Epilobium_tetragonum	2018	H SCAP	PALEOTEMP	7	7	5	5	5	5	x			x
Equisetum_arvense	2018	G RHIZ	CIRCUMBOR	6	7	5	6	6	3	x			
Equisetum_ramosissimum	2018	G RHIZ	CIRCUMBOR	7	7	6	3	7	1	x			
Eragrostis_frankii	2018	T SCAP	AVV NATURALIZZ	8	8	5	4	6	3				x
Eragrostis_mexicana	2018	T SCAP	AVV NATURALIZZ	8	8	5	4	6	3				x
Eragrostis_pectinacea	2018	T SCAP	AVV NATURALIZZ	8	8	5	4	6	3	x	x	x	x
Eragrostis_pilosa	2018	T SCAP	COSMOPOL	8	8	5	3	6	2	x	x	x	x
Erigeron_annuus	2018	T SCAP	AVV NATURALIZZ	7	7	5	6	5	4	x	x	x	x
Erigeron_bonariensis	2018	T SCAP	AVV NATURALIZZ	8	8	5	3	6	7	x	x	x	x
Erigeron_canadensis	2018	T SCAP	AVV NATURALIZZ	8	6	5	5	6	7	x	x	x	x
Erigeron_karvinskianus	2018	H SCAP	SUBTROP	7	8	5	3	3	2			x	x

Erigeron_sumatrensis	2018	T SCAP	AVV NATURALIZZ	8	8	5	3	6	7	x	x	x	x
Eriobotrya_japonica	2018	P SCAP	COLTIV	9	9	5	5	5	5	x		x	x
Erodium_cicutarium	2018	T SCAP	SUBCOSMOP	8	7	5	3	5	3				x
Erodium_moschatum	2018	T SCAP	EURIMEDIT	11	9	5	2	5	2	x			
Erophila_verna	2018	T SCAP	CIRCUMBOR	9	7	4	2	4	1	x		x	
Euonymus_europaeus	2018	P CAESP	EURASIAT	6	5	5	5	8	5	x			
Eupatorium_cannabinum	2018	H SCAP	PALEOTEMP	7	7	5	7	5	7		x		
Euphorbia_amygdaloides	2018	CH	EUROP-CAUCAS	4	5	4	5	7	6	x			
Euphorbia_characias	2018	NP	STENOMEDIT	8	10	4	2	6	1	x			
Euphorbia_helioscopia	2018	T SCAP	COSMOPOL	9	7	5	3	5	6	x	x	x	
Euphorbia_hirsuta	2018	G RHIZ	STENOMEDIT	7	7	4	6	7	4	x			
Euphorbia_lathyris	2018	H BIENNE	EURIMEDIT-TURAN	6	8	6	3	5	6	x	x		
Euphorbia_maculata	2018	T SCAP	AVV NATURALIZZ	7	8	5	2	5	4	x	x	x	x
Euphorbia_peplus	2018	T SCAP	EUROSIB	6	7	4	4	5	7	x	x	x	x
Euphorbia_prostrata	2018	T REPT	AVV NATURALIZZ	7	8	5	2	5	4	x	x	x	x
Fallopia_convolvulus	2018	T SCAP	CIRCUMBOR	8	7	4	4	5	3	x	x	x	x
Fallopia_multiflora	2018	P LIAN	COLTIV	6	7	7	6	6	6	x			
Festuca_pratensis	2018	H CAESP	EURASIAT	8	6	6	5	5	6	x		x	
Festuca_rubra	2018	H CAESP	CIRCUMBOR	8	4	5	4	4	3	x		x	x
Ficus_carica	2018	P SCAP	EURIMEDIT-TURAN	7	8	6	4	5	5	x	x	x	x
Foeniculum_vulgare	2018	H SCAP	S-EURIMEDIT	9	8	5	3	7	7	x			
Fontanesia_phillyraeoides	2018	P CAESP	E-STENOMEDIT	11	10	5	3	6	2	x			
Fragaria_vesca	2018	CH REPT	EUROSIB	6	7	4	4	6	5	x	x	x	
Fraxinus_excelsior	2018	P SCAP	EUROP-CAUCAS	4	5	4	7	7	7				x
Fraxinus_sp.	2018	P SCAP	COLTIV	4	5	4	7	7	7	x		x	x
Fumaria_capreolata	2018	T SCAP	EURIMEDIT	7	9	5	3	5	3				x
Fumaria_officinalis	2018	T SCAP	SUB COSMOPOL	7	7	5	4	5	6	x			x
Galanthus_nivalis	2018	G BULB	EUROP-CAUCAS	5	7	4	4	7	7	x			
Galinsoga_parviflora	2018	T SCAP	AVV NATURALIZZ	7	6	5	7	5	8				x
Galinsoga_quadriradiata	2018	T SCAP	AVV NATURALIZZ	7	6	5	7	5	8	x	x		x
Galium_album	2018	H SCAP	EURIMEDIT	6	5	5	5	5	4	x			
Galium_aparine	2018	T SCAP	EURASIAT	6	7	5	4	5	5	x	x	x	x
Galium_divaricatum	2018	T SCAP	STENOMEDIT	11	9	4	2	6	1	x			x

Galium_murale	2018	T SCAP	STENOMEDIT	11	9	4	2	6	1	x			
Galium_parisiense	2018	T SCAP	EURIMEDIT	11	8	5	2	3	1	x	x	x	x
Galium_verum	2018	T SCAP	EUROP-CAUCAS	7	6	6	4	7	3	x			
Geranium_dissectum	2018	T SCAP	EURASIAT	7	8	5	2	5	2	x	x	x	
Geranium_molle	2018	T SCAP	EURASIAT	7	6	5	3	5	4	x	x	x	x
Geranium_nodosum	2018	G RHIZ	N-MEDIT-MONT	4	4	4	6	6	6	x			
Geranium_purpureum	2018	T SCAP	EURIMEDIT	7	8	5	3	6	3	x	x	x	x
Geranium_rotundifolium	2018	T SCAP	PALEOTEMP	7	8	5	3	6	3	x		x	x
Geum_urbanum	2018	H SCAP	CIRCUMBOR	4	5	5	5	6	7	x	x	x	
Ginkgo_biloba	2018	P SCAP	COLTIV	7	6	7	2	6	2	x			
Glechoma_hederacea	2018	CH REPT	CIRCUMBOR	6	7	4	4	5	3	x		x	
Gleditsia_triacanthos	2018	P CAESP	AVV NATURALIZZ	8	7	5	4	5	5	x		x	
Gnaphalium_pensylvanicum	2018	H SCAP	AVV NATURALIZZ	9	8	6	3	5	3			x	
Hedera _algeriensis	2018	P LIAN	COLTIV	4	6	4	5	6	5	x			
Hedera_helix	2018	P LIAN	SUB MEDIT-SUB ATLANT	4	5	4	5	6	5	x	x	x	x
Hedysarum_coronarium	2018	H SCAP	W-STENOMEDIT	7	8	4	5	7	3			x	
Helianthus_tuberosus	2018	G BULB	AVV NATURALIZZ	8	7	5	7	6	6	x			
Heliotropium_amplexicaule	2018	CH FRUT	AVV NATURALIZZ	9	9	5	3	5	2	x			
Heliotropium_europaeum	2018	T SCAP	EURIMEDIT	11	8	5	3	7	2	x			x
Helleborus_foetidus	2018	CH SUFFR	SUBATLANT	5	6	4	4	8	3	x			
Helleborus_viridis	2018	G RHIZ	SUBATLANT	3	6	4	5	8	5	x			
Helminthotheca_echioides	2018	T SCAP	EURIMEDIT	11	8	5	2	6	2	x		x	x
Hibiscus_syriacus	2018	P CAESP	AVV NATURALIZZ	9	8	5	3	6	5	x		x	x
Hordeum_murinum ssp.	2018	T SCAP	EURIMEDIT	9	9	5	3	5	3	x	x	x	x
Hordeum_murinum	2018	T SCAP	CIRCUMBOR	8	8	4	5	5	3	x	x	x	x
Humulus_lupulus	2018	P LIAN	EUROP-CAUCAS	7	6	4	8	6	8	x			
Hyacinthoides_hispanica	2018	G BULB	W-STENOMEDIT	7	7	4	4	5	4	x			
Hyacinthus_orientalis	2018	G BULB	AVV NATURALIZZ	7	7	5	3	5	5	x			
Hylotelephium_telephium	2018	H SCAP	COLTIV	7	6	4	4	7	5	x			x
Hypericum_perforatum	2018	H SCAP	PALEOTEMP	7	8	6	4	6	5	x	x		
Hypochaeris_radicata	2018	H ROS	EUROP-CAUCAS	9	8	4	2	6	1	x		x	x
Iberis_semperflorens	2018	CH SUFFR	COLTIV	6	8	3	3	6	2			x	
Ilex_aquifolium	2018	P CAESP	EURIMEDIT	4	5	4	5	4	5	x			

Iris_foetidissima	2018	G RHIZ	EURIMEDIT	7	7	5	4	4	5	x		x	
Iris_germanica	2018	G RHIZ	AVV NATURALIZZ	7	7	5	3	5	4	x			
Juglans_nigra	2018	P SCAP	COLTIV	6	6	5	5	6	6	x			
Juglans_regia	2018	P SCAP	AVV NATURALIZZ	6	6	6	5	6	6	x		x	
Kalanchoe _daigremontiana	2018	CH SUCC	COLTIV	10	10	5	2	5	1	x		x	x
Koelreuteria_paniculata	2018	P SCAP	COLTIV	7	8	5	4	5	4	x			
Laburnum_anagyroides	2018	P CAESP	S-EUROP-SUDSIB	5	6	6	5	7	7	x			
Lactuca_saligna	2018	T SCAP	EURIMEDIT-TURAN	11	7	7	4	6	4	x	x	x	x
Lactuca_serriola	2018	H BIENNE	S-EUROP-SUDSIB	9	7	7	4	6	4	x	x	x	x
Lamium_amplexicaule	2018	T SCAP	PALEOTEMP	7	7	5	4	5	7	x			x
Lamium_maculatum	2018	H SCAP	EURASIAT	7	7	5	4	5	4	x			
Lamium_purpureum	2018	T SCAP	EURASIAT	7	7	5	4	5	5	x	x	x	x
Lapsana_communis	2018	T SCAP	PALEOTEMP	5	7	5	5	6	7	x		x	
Lathyrus_sylvestris	2018	H SCAND	EUROP-CAUCAS	7	5	6	4	4	4	x			
Laurus_nobilis	2018	P CAESP	STENOMEDIT	2	7	4	8	4	6	x		x	x
Lepidium_coronopus	2018	T REPT	SUB COSMOPOL	8	8	5	3	4	2	x			
Lepidium_graminifolium	2018	H SCAP	EURIMEDIT	8	8	5	3	6	3	x			
Lepidium_virginicum	2018	T SCAP	AVV NATURALIZZ	8	6	5	4	6	7	x			x
Leucanthemum_maximum	2018	H SCAP	COLTIV	7	6	5	4	7	2	x			
Ligustrum_japonicum	2018	P CAESP	COLTIV	7	8	5	4	5	4	x		x	
Ligustrum_lucidum	2018	P CAESP	AVV NATURALIZZ	7	8	5	4	5	4	x		x	x
Ligustrum_sinense	2018	P CAESP	AVV NATURALIZZ	7	8	5	4	5	4	x		x	x
Ligustrum_vulgare	2018	NP	EUROP-CAUCAS	7	6	4	4	8	5	x			
Linaria_vulgaris	2018	H SCAP	EURASIAT	8	5	5	3	7	3	x			
Liquidambar styraciflua	2018	P SCAP	COLTIV							x			
Lolium_multiflorum	2018	T SCAP	CIRCUMBOR	7	7	5	4	6	6	x			
Lolium_perenne	2018	H CAESP	CIRCUMBOR	8	5	4	5	6	7	x	x	x	x
Lonicera_japonica	2018	P LIAN	AVV NATURALIZZ	7	8	5	3	6	4	x			
Lonicera_ligustrina	2018	P CAESP	COLTIV	6	5	6	6	5	5	x			
Lotus_corniculatus	2018	H SCAP	PALEOTEMP	7	7	5	4	7	2	x			
Lotus_tenuis	2018	H SCAP	PALEOTEMP	9	7	5	6	7	7	x			
Lunaria_annua	2018	H SCAP	SE-EUROP	4	6	6	6	7	6	x		x	x
Maclura_pomifera	2018	P SCAP	AVV NATURALIZZ	8	7	5	5	5	5	x			

Magnolia_grandiflora	2018	P SCAP	COLTIV	8	7	4	3	7	5	x			
Mahonia_aquifolium	2018	NP	COLTIV	7	7	4	5	4	5	x		x	x
Mahonia_japonica	2018	NP	COLTIV	7	7	4	5	4	5	x		x	x
Malus_domestica	2018	P SCAP	AVV NATURALIZZ	7	7	5	5	5	5	x	x	x	
Malva_sylvestris	2018	H SCAP	EUROSIB	8	6	4	4	6	8	x	x	x	x
Medicago_arabica	2018	T SCAP	EURIMEDIT	9	9	5	2	6	2	x			x
Medicago_lupulina	2018	T SCAP	PALEOTEMP	7	5	5	4	8	7	x	x		x
Medicago_minima	2018	T SCAP	EURIMEDIT	11	7	5	3	8	1	x		x	
Medicago_sativa	2018	T SCAP	EURASIAT	8	5	7	3	9	3	x			
Melia_azedarach	2018	P SCAP	COLTIV	7	8	5	5	5	4	x			
Melica_uniflora	2018	H CAESP	PALEOTEMP	3	5	5	5	6	5	x			
Melilotus_officinalis	2018	H BIENNE	EURASIAT	8	5	6	3	8	7	x			
Mentha_spicata	2018	H SCAP	EURIMEDIT	7	6	5	8	8	6	x	x		x
Mercurialis_annua	2018	T SCAP	PALEOTEMP	7	7	5	4	7	8	x			x
Mirabilis_jalapa	2018	G BULB	AVV NATURALIZZ	6	7	5	4	6	6	x		x	x
Morus_alba	2018	P SCAP	AVV NATURALIZZ	8	7	5	5	5	5	x		x	
Muscari_comosum	2018	G BULB	EURIMEDIT	7	8	5	3	7	0	x			
Muscari_neglectum	2018	G BULB	EURIMEDIT	7	7	5	4	6	3	x			
Myosotis_arvensis	2018	T SCAP	EUROP-CAUCAS	6	5	5	5	6	6	x		x	x
Myosotis_ramosissima	2018	T SCAP	EUROP-CAUCAS	9	8	5	2	4	3	x			
Nandina _domestica	2018	NP	COLTIV	7	7	4	4	5	4	x	x	x	
Narcissus_ex-cv- gruppo 2	2018	G BULB	COLTIV	8	8	4	4	5	4	x			
Nerium_oleander	2018	P CAESP	S-STENOMEDIT	11	11	5	7	6	3	x	x	x	
Ocimum_basilicum	2018	H SCAP	AVV NATURALIZZ	7	8	5	6	5	7			x	
Olea_europaea	2018	CH PULV	S-STENOMEDIT	11	10	4	1	6	2	x			x
Ophiopogon_japonicus	2018	G RHIZ	COLTIV	9	8	5	6	5	5	x			
Orchis_purpurea	2018	G BULB	EURASIAT	5	7	5	4	8	5			x	
Ornithogalum_umbellatum	2018	G BULB	EURIMEDIT	5	6	5	5	7	5	x		x	
Orobanche_hederae	2018	T SCAP	EURIMEDIT	6	7	5	4	5	5	x			
Ostrya_carpinifolia	2018	P CAESP	CIRCUMBOR	4	8	4	4	6	5	x		x	
Oxalis_articulata	2018	G RHIZ	COLTIV	8	9	4	3	4	5	x	x	x	x
Oxalis_corniculata	2018	CH REPT	EURIMEDIT	7	7	0	4	6	6	x	x	x	x
Oxalis_dillenii	2018	H SCAP	AVV NATURALIZZ	7	7	5	5	5	7	x	x	x	x

Oxalis_pes-caprae	2018	G BULB	AVV NATURALIZZ	8	10	4	3	6	5				x
Papaver_dubium	2018	T SCAP	EURIMEDIT-TURAN	6	6	6	4	5	5	x			
Papaver_rhoeas	2018	T SCAP	EURIMEDIT	6	6	5	5	7	5	x		x	x
Parietaria_judaica	2018	H SCAP	EURIMEDIT-MACARON	7	8	5	3	6	6	x	x	x	x
Parietaria_officinalis	2018	H SCAP	EUROP-CAUCAS	4	8	4	5	7	7	x	x	x	x
Parthenocissus_quinquefolia	2018	P LIAN	AVV NATURALIZZ	5	7	5	5	5	5	x		x	x
Passiflora_coerulea	2018	P LIAN	AVV NATURALIZZ	6	6	5	5	5	5	x		x	
Paulownia_tomentosa	2018	P CAESP	AVV NATURALIZZ	6	7	5	5	5	2	x			
Petasites_fragrans	2018	G RHIZ	EURIMEDIT	7	8	5	7	7	6	x			
Petunia_hybrida	2018	T SCAP	COLTIV	10	10	4	4	6	4			x	
Philadelphus_coronarius	2018	NP	SUB ENDEM	8	6	4	4	7	4	x			
Phalaris_brachystachys	2018	T SCAP	STENOMEDIT	7	7	5	5	6	4	x			
Phoenix_canariensis	2018	P SCAP	COLTIV	11	10	2	4	6	4				x
Phragmites_australis	2018	G RHIZ	SUBCOSMOP	7	5	5	10	7	5	x			
Phytolacca_americana	2018	G RHIZ	AVV NATURALIZZ	9	8	5	5	5	4	x	x	x	x
Picris_hieracioides	2018	H SCAP	EUROSIB	8	7	5	4	8	4	x	x	x	x
Pinus_pinea	2018	P SCAP	EURIMEDIT	11	8	5	2	4	3	x			x
Pistacia_terebinthus	2018	P CAESP	EURIMEDIT	9	8	5	2	7	2	x			
Pisum_sativum	2018	P SCAP	COLTIV	8	7	5	4	5	7				x
Pittosporum_tobira	2018	P CAESP	COLTIV	10	9	5	2	6	2	x			
Plantago_coronopus	2018	T SCAP	EURIMEDIT	8	7	5	7	7	4				x
Plantago_lanceolata	2018	H ROS	EURASIAT	6	7	5	4	6	5	x	x	x	x
Plantago_major	2018	H ROS	SUB COSMOPOL	8	7	5	5	6	7	x	x	x	x
Platanus_x hispanica	2018	P SCAP	EURIMEDIT	9	7	5	8	6	6	x	x	x	x
Platycladus_orientalis	2018	P SCAP	COLTIV	7	7	6	3	6	3			x	
Poa_annua	2018	T CAESP	COSMOPOL	7	7	5	6	6	8	x	x	x	x
Poa_bulbosa	2018	H CAESP	PALEOTEMP	8	8	7	2	4	1	x			x
Poa_pratensis	2018	H CAESP	CIRCUMBOR	6	7	5	5	6	5	x		x	x
Poa_trivialis	2018	H CAESP	EURASIAT	6	7	5	7	6	7	x	x	x	x
Polycarpon_tetraphyllum	2018	T SCAP	EURIMEDIT	7	7	5	4	5	6	x	x	x	x
Polygonum_aviculare	2018	T REPT	COSMOPOL	7	7	5	3	6	1	x	x	x	x
Polypodium_interjectum	2018	H ROS	CIRCUMBOR	3	7	5	3	3	5	x	x		
Poncirus_trifoliata	2018	P CAESP	COLTIV	9	9	5	2	3	5	x			

Populus_alba	2018	P SCAP	PALEOTEMP	5	8	7	5	8	6	x	x		
Populus_nigra	2018	P SCAP	PALEOTEMP	5	7	6	8	7	7	x			x
Portulaca_oleracea	2018	T SCAP	SUB COSMOPOL	7	8	5	4	7	7	x	x	x	x
Potentilla_indica	2018	H ROS	AVV NATURALIZZ	5	7	5	6	5	7	x	x	x	x
Potentilla_reptans	2018	H ROS	PALEOTEMP	6	6	5	6	7	5	x	x	x	x
Primula_vulgaris	2018	H ROS	EUROP-CAUCAS	6	5	4	5	7	5	x	x		x
Prunus_armeniaca	2018	P CAESP	COLTIV	9	7	6	5	5	5	x			
Prunus_avium	2018	P SCAP	PONTICA	4	5	6	5	7	5	x	x	x	x
Prunus_cerasifera	2018	P CAESP	AVV NATURALIZZ	9	7	5	5	5	5	x	x	x	x
Prunus_cerasus	2018	P SCAP	PONTICA	9	7	6	5	5	5	x			
Prunus_dulcis	2018	P SCAP	COLTIV	9	7	5	3	5	3	x			
Prunus_laurocerasus	2018	P CAESP	COLTIV	9	7	5	5	5	5	x			
Prunus_persica	2018	P SCAP	COLTIV	9	7	5	5	5	5		x	x	
Prunus_pissardii	2018	P CAESP	COLTIV	9	7	6	5	5	5	x			x
Prunus_spinosa	2018	P CAESP	EUROP-CAUCAS	7	5	5	4	6	5	x			
Pteris_multifida	2018	G RHIZ	AVV NATURALIZZ	6	8	3	4	4	2	x			
Pteris_vittata	2018	H ROS	PANTROP	6	11	3	4	4	2	x			
Pulmonaria_officinalis	2018	H SCAP	CENTRO-EUROP	5	6	5	5	8	6	x			
Punica_granatum	2018	P SCAP	AVV NATURALIZZ	9	8	5	4	6	4	x			
Pyracantha_rogersiana	2018	P CAESP	AVV NATURALIZZ	5	8	4	3	5	3	x			
Pyrus_communis	2018	P SCAP	AVV NATURALIZZ	7	7	5	5	5	5	x			
Quercus_cerris	2018	P SCAP	N-EURIMEDIT	6	8	5	4	4	4	x			
Quercus_ilex	2018	P SCAP	STENOMEDIT	2	9	4	3	6	5	x		x	x
Quercus_pubescens	2018	P CAESP	SE-EUROP	7	8	6	3	7	4	x		x	x
Quercus_robur	2018	P SCAP	EUROCAUCAS	7	6	6	6	5	6	x		x	x
Ranunculus _repens	2018	H REPT	COSMOPOL	6	7	5	7	6	7	x		x	
Ranunculus_acris	2018	H SCAP	SUBCOSMOP	7	7	5	4	6	5	x		x	x
Ranunculus_bulbosus	2018	H SCAP	EURASIAT	8	6	5	3	7	3	x		x	x
Ranunculus_ficaria	2018	G BULB	EURASIAT	11	7	5	5	5	6	x	x	x	x
Ranunculus_neapolitanus	2018	H SCAP	NE-MEDIT-MONT	8	6	5	3	7	3	x	x	x	x
Ranunculus_parviflorus	2018	T SCAP	EURIMEDIT-SUBATL	8	7	4	5	6	5	x	x	x	x
Ranunculus_sardous	2018	T SCAP	EURIMEDIT	8	7	5	8	6	7	x		x	x
Robinia_pseudoacacia	2018	P CAESP	AVV NATURALIZZ	5	7	5	4	6	8	x		x	x

Rorippa_sylvestris	2018	H SCAP	EURASIAT	6	6	5	8	8	6	x	x	x	x
Rosa_canina	2018	NP	PALEOTEMP	8	5	5	4	6	5	x			
Rosmarinus_officinalis	2018	NP	STENOMEDIT	11	8	4	2	6	1			x	
Rostraria_cristata	2018	T SCAP	SUBCOSMOP	7	5	5	6	8	2	x			
Rubus_caesius	2018	NP	EURASIAT	7	5	5	7	7	9	x			
Rubus_ulmifolius	2018	NP	EURIMEDIT	5	8	5	4	5	8	x		x	x
Rumex_crispus	2018	H SCAP	SUBCOSMOP	7	5	5	6	6	5	x	x	x	x
Rumex_pulcher	2018	H SCAP	EURIMEDIT	8	8	5	2	6	9	x		x	x
Ruscus_aculeatus	2018	CH FRUT	EURIMEDIT	4	8	5	4	5	5	x			
Sagina_apetala	2018	T SCAP	EURIMEDIT	8	7	5	6	4	5	x	x	x	x
Sagina_procumbens	2018	H CAESP	SUBCOSMOP	6	7	5	6	7	6	x	x	x	x
Salix_alba	2018	P SCAP	PALEOTEMP	5	6	6	7	8	7		x	x	
Salix_caprea	2018	P CAESP	EURASIAT	7	4	5	6	7	4				x
Salix_cinerea	2018	P CAESP	PALEOTEMP	7	4	5	9	5	4		x		
Salpichroa_origanifolia	2018	CH FRUT	AVV NATURALIZZ	6	7	5	5	5	5	x			
Salvia_pratensis	2018	H SCAP	EURIMEDIT	8	6	6	4	8	4	x			x
Salvia_verbenaca	2018	H SCAP	STENOMEDIT-ATL	8	8	4	3	5	7	x		x	
Sambucus_ebulus	2018	G RHIZ	EURIMEDIT	8	6	5	5	8	7	x			
Sambucus_nigra	2018	P CAESP	EUROP-CAUCAS	7	5	4	5	6	9	x		x	x
Sanguisorba_minor	2018	H SCAP	PALEOTEMP	7	6	5	3	8	2	x			
Saxifraga_Stolonifera	2018	H ROS	EURIMEDIT	4	9	5	3	4	5	x			
Saxifraga_tridactylites	2018	T SCAP	EURIMEDIT	8	6	5	2	7	1	x	x	x	x
Scilla_bifolia	2018	G BULB	EUROP-CAUCAS	5	6	5	6	7	6	x			
Sedum_acre	2018	CH SUCC	EUROP-CAUCAS	8	5	4	1	6	1	x			x
Sedum_album	2018	CH SUCC	EURIMEDIT	11	7	5	2	6	1	x			
Sedum_dasyphyllum	2018	CH SUCC	EURIMEDIT	7	7	5	3	6	5	x	x		
Sedum_hispanicum	2018	T SCAP	SE-EUROP	9	6	6	1	7	1			x	
Sedum lineare	2018	CH SUCC	COLTIV							x			
Sedum_palmeri	2018	CH SUCC	COLTIV	9	9	5	2	6	1		x	x	x
Sedum_pseudorupestre	2018	CH SUCC	MEDIT-MONT	7	5	4	2	4	1	x		x	
Senecio_inaequidens	2018	T SCAP	AVV NATURALIZZ	9	7	5	2	5	1	x			
Senecio_vulgaris	2018	T SCAP	EURIMEDIT	7	7	5	5	6	8				x
Setaria_pumila	2018	T SCAP	SUBCOSMOP	7	7	5	4	5	6	x	x	x	x

Setaria_verticillata	2018	T SCAP	SUBTROP	7	8	5	4	6	8	x	x	x	x
Setaria_viridis	2018	T SCAP	SUBCOSMOP	7	6	5	4	6	7	x	x	x	x
Silene_flos-cuculi	2018	H SCAP	EUROSIB	7	5	4	6	6	6	x			
Silene_latifolia	2018	H BIENNE	PALEOTEMP	8	7	5	4	6	7	x			
Sinapis_arvensis	2018	T SCAP	STENOMEDIT	7	5	4	4	8	6			x	x
Sisymbrium_irio	2018	T SCAP	PALEOTEMP	8	8	5	3	7	5		x		x
Sisymbrium_officinale	2018	T SCAP	PALEOTEMP	8	6	5	4	6	7	x		x	
Solanum_dulcamara	2018	NP	PALEOTEMP	7	5	5	8	6	8	x			
Solanum_lycopersicum	2018	T SCAP	AVV NATURALIZZ	7	7	5	5	5	7	x	x	x	x
Solanum_nigrum	2018	T SCAP	COSMOPOL	7	6	5	3	5	7	x	x	x	x
Solanum _pseudocapsicum	2018	CH	COLTIV	7	6	5	5	5	6			x	x
Solanum_villosum	2018	T SCAP	EURIMEDIT	7	6	5	3	5	7	x		x	
Soleirolia_soleirolii	2018	H SCAP	W-MEDIT-NE-SIC	5	10	3	3	4	4	x			
Sonchus_asper	2018	T SCAP	STENOMEDIT	7	5	5	4	7	7	x	x	x	x
Sonchus_oleraceus	2018	T SCAP	EURASIAT	7	5	5	4	8	8	x	x	x	x
Sonchus_tenerrimus	2018	T SCAP	STENOMEDIT	7	8	4	2	5	4	x	x	x	x
Sophora_japonica	2018	P SCAP	COLTIV	6	7	5	4	5	8	x		x	x
Sorghum_halepense	2018	G RHIZ	COSMOP	8	8	5	7	8	8	x		x	x
Stachys_annua	2018	T SCAP	EURIMEDIT	7	6	5	3	8	4	x			
Stellaria_aquatica	2018	H SCAP	EUROSIB	7	5	4	8	6	8				x
Stellaria_media	2018	T REPT	COSMOPOL	6	7	5	4	7	8	x	x	x	x
Sternbergia_lutea	2018	G BULB	MEDIT-MONT	6	6	3	4	5	4	x			
Symphoricarpos_x chenaultii	2018	P CAESP	AVV NATURALIZZ	8	7	5	5	5	5	x			
Symphyotrichum_lanceolatum	2018	H SCAP	AVV NATURALIZZ	7	7	6	6	6	8	x	x	x	x
Symphyotrichum_squamatum	2018	T SCAP	AVV NATURALIZZ	8	8	5	4	7	7	x		x	x
Symphytum_bulbosum	2018	G RHIZ	SE-EUROP	4	7	6	4	5	3	x			
Symphytum_officinale	2018	H SCAP	EUROP-CAUCAS	7	6	4	8	6	8	x			
Symphytum_orientale	2018	H SCAP	AVV NATURALIZZ	4	8	5	3	5	3	x	x		
Symphytum_tuberosum	2018	G RHIZ	SE-EUROP	4	5	6	6	7	5	x	x	x	
Syringa_vulgaris	2018	P CAESP	OROF SE-EUROP	6	7	5	5	5	4	x			
Taraxacum_officinale	2018	H ROS	CIRCUMBOR	7	7	5	5	6	7	x	x	x	x
Taxus_baccata	2018	P SCAP	PALEOTEMP	2	6	5	5	7	0	x		x	
Thelypteris_palustris	2018	G RHIZ	SUBCOSMOP	5	7	5	8	5	6	x			

Thlaspi_arvense	2018	T SCAP	AVV NATURALIZZ	6	5	5	5	7	7	x			
Thymus_serpyllum	2018	CH FRUT	W-STENOMEDIT	8	8	4	2	7	1	x			
Tilia_americana	2018	P CAESP	COLTIV	4	5	4	4	6	5	x	x	x	
Torilis_arvensis	2018	T SCAP	SUB COSMOPOL	7	8	5	4	7	6	x			x
Torilis_nodosa	2018	T SCAP	EURIMEDIT-TURAN	7	8	6	4	7	6	x	x	x	x
Trachycarpus_fortunei	2018	P SCAP	COLTIV	7	7	6	3	6	3	x		x	x
Tribulus_terrestris	2018	T REPT	COSMOPOL	8	8	6	2	5	3	x			
Trifolium_campestre	2018	T SCAP	PALEOTEMP	8	5	5	4	6	3	x			
Trifolium_fragiferum	2018	CH REPT	PALEOTEMP	8	6	5	7	8	7	x		x	x
Trifolium_pratense	2018	CH REPT	SUB COSMOPOL	7	7	4	4	6	5	x		x	x
Trifolium_repens	2018	CH REPT	PALEOTEMP	8	7	5	4	6	7	x	x	x	x
Trifolium_scabrum	2018	T REPT	EURIMEDIT	11	8	5	2	9	1	x	x		x
Trisetaria_flavescens	2018	H CAESP	EURASIAT	7	7	5	4	6	5	x			
Triticum_aestivum	2018	T SCAP	COLTIV	8	8	5	5	5	5	x		x	x
Tulipa_gesneriana	2018	G RHIZ	COLTIV	7	7	5	3	6	6	x			
Tussilago_farfara	2018	G RHIZ	PALEOTEMP	8	7	5	6	8	7	x	x	x	
Ulmus_minor	2018	P CAESP	EUROP-CAUCAS	5	7	5	4	8	5	x		x	x
Ulmus_pumila	2018	P SCAP	COLTIV	5	7	5	4	9	5	x	x	x	x
Umbilicus_rupestris	2018	G BULB	STENOMEDIT-ATL	5	8	4	3	6	3	x	x	x	x
Urtica_dioica	2018	H SCAP	SUBCOSMOP	7	7	5	6	6	8	x			x
Urtica_urens	2018	T SCAP	SUBCOSMOP	7	6	5	5	7	8				x
Verbascum_blattaria	2018	H BIENNE	PALEOTEMP	8	6	7	3	7	6	x			x
Verbascum_phlomoides (cfr.)	2018	H BIENNE	EURIMEDIT	7	8	5	3	7	7	x			
Verbascum_sinuatum	2018	H BIENNE	EURIMEDIT	9	8	5	3	7	7	x			
Verbena_officinalis	2018	H SCAP	PALEOTEMP	9	5	5	4	6	6	x	x	x	x
Veronica_arvensis	2018	T SCAP	PALEOTEMP	5	5	5	5	6	5	x	x	x	x
Veronica_chamaedrys	2018	H SCAP	S-EUROP-SUDSIB	6	7	6	4	6	5	x		x	
Veronica_cymbalaria	2018	T SCAP	EURIMEDIT	7	7	5	4	3	2	x			
Veronica_hederifolia	2018	T SCAP	EURASIAT	6	6	5	5	3	7	x	x	x	x
Veronica_peregrina	2018	T SCAP	AVV NATURALIZZ	7	7	5	6	5	5	x			x
Veronica_persica	2018	T SCAP	AVV NATURALIZZ	8	7	5	5	5	6	x	x	x	x
Veronica_polita	2018	T SCAP	PALEOTEMP	5	6	5	4	8	7	x		x	
Viburnum_tinus	2018	P CAESP	STENOMEDIT	5	9	4	4	5	3	x		x	

Vicia_sativa	2018	T SCAP	MEDIT-TURAN	5	5	6	4	6	5	x		x	
Vinca_major	2018	CH REPT	EURIMEDIT	6	7	5	4	5	3	x			
Vinca_minor	2018	CH REPT	EUROP-CAUCAS	4	6	4	5	6	6	x			
Viola_alba	2018	H ROS	EURIMEDIT	5	8	5	5	7	6	x			
Viola_odorata	2018	H ROS	EURIMEDIT	5	6	5	5	6	8	x	x	x	x
Viola_cfr. riviniana	2018	H SCAP	EUROP-CAUCAS	5	7	5	5	3	5	x		x	x
Vitis_labrusca	2018	P LIAN	COLTIV	6	8	5	6	8	6	x			
Vitis_vinifera	2018	P LIAN	COLTIV	6	8	5	6	8	6	x		x	x
Vulpia_myuros	2018	T CAESP	SUBCOSMOP	8	9	5	2	6	2	x		x	x
Wisteria_sinensis	2018	P LIAN	COLTIV	6	8	5	6	8	6	x		x	
Xanthium_orientale	2018	T SCAP	AVV NATURALIZZ	8	8	5	5	6	1	x			

DATASET: "Gabelli, 1894"

Acanthus_mollis	1894	H SCAP	W-STENOMEDIT	7	8	4	3	5	4	x			
Achillea _millefolium	1894	H SCAP	EUROSIB	8	X	X	4	X	5				
Aegopodium_podagraria	1894	G RHIZ	EUROSIB	5	X	4	6	7	8				
Ailanthus_altissima	1894	P SCAP	AVV NATURALIZZ	6	7	5	5	5	5				
Ajuga_chamaepitys	1894	T SCAP	EURIMEDIT	7	8	5	4	9	2				
Alcea_rosea	1894	H SCAP	COLTIV	9	8	5	3	6	4	x			
Alopecurus_myosuroides	1894	T SCAP	SUBCOSMOP	6	6	5	6	7	7				
Amaranthus_albus	1894	T SCAP	AVV NATURALIZZ	9	9	6	3	X	7		x	x	x
Amaranthus_deflexus	1894	T SCAP	AVV NATURALIZZ	8	8	5	4	6	9				
Amaranthus_viridis	1894	T SCAP	AVV NATURALIZZ	8	8	5	4	6	8				
Anagallis_arvensis	1894	T REPT	SUBCOSMOP	8	7	5	4	9	5				
Anagallis_arvensis	1894	T REPT	EURIMEDIT	6	6	5	5	X	6				
Anthoxanthum_odoratum	1894	H CAESP	EURASIAT	X	X	5	X	5	3				
Antirhinum _majus	1894	CH FRUT	W-STENOMEDIT	11	8	4	2	X	1	x	x	x	x
Arabidopsis_thaliana	1894	T SCAP	PALEOTEMP	6	X	5	4	5	4				
Arenaria_serpyllifolia ssp.	1894	T SCAP	SUBCOSMOP	9	5	X	4	X	X	x	x	x	x
Arenaria_serpyllifolia	1894	T SCAP	SUBCOSMOP	9	5	X	5	X	X	x	x	x	x
Asplenium_rutamuraria	1894	H ROS	CIRCUMBOR	8	X	4	3	8	2		x		x
Asplenium_trichomanes	1894	H ROS	COSMOP TEMP	5	X	5	5	X	4		x		x

Avena_barbata	1894	T SCAP	EURIMEDIT	8	8	5	3	7	2				
Ballota_nigra	1894	H SCAP	EURIMEDIT	8	6	5	5	X	8				
Bassia_scoparia	1894	T SCAP	AVV NATURALIZZ	9	6	9	3	2	1				x
Bellis_perennis	1894	H ROS	EUROP-CAUCAS	9	5	4	X	X	5	x	x	x	x
Borrago_officinalis	1894	T SCAP	EURIMEDIT	7	8	5	3	5	5	x			
Bromus_sterilis	1894	T SCAP	EURIMEDIT	7	7	5	4	X	5	x	x	x	x
Buglossoides_arvensis	1894	T SCAP	EURIMEDIT	5	X	5	X	7	5	x			
Capparis_spinosa	1894	NP	EURASIAT	9	10	5	2	5	1			x	x
Capsella_bursa-pastoris	1894	H BIENNE	COSMOPOL	7	X	5	5	5	4	x			
Cardamine_hirsuta	1894	T SCAP	COSMOPOL	7	8	5	3	5	4	x	x	x	x
Catapodium_rigidum	1894	T SCAP	EURIMEDIT	8	8	5	2	5	4				
Centaurea_nigriscens	1894	H SCAP	ENDEM ALP	7	6	5	4	5	4				
Cerastium_glomeratum	1894	T SCAP	EURIMEDIT	7	X	5	5	5	5				
Cerastium_ligusticum	1894	T SCAP	W-STENOMEDIT	11	9	4	2	3	1				
Chelidonium_majus	1894	H SCAP	EURASIAT	6	6	X	5	X	8		x		
Chenopodium_album	1894	T SCAP	SUBCOSMOP	7	7	5	4	5	7	x	x	x	x
Chenopodium_vulvaria	1894	T SCAP	EURIMEDIT	7	7	5	4	X	9				
Cichorium_intybus	1894	H SCAP	PALEOTEMP	9	6	5	3	8	5	x			
Cirsium_arvense	1894	G RAD	EURASIAT	8	X	X	4	X	7	x	x	x	x
Citrullus_vulgaris	1894	H SCAND	S-STENOMEDIT	11	12	5	1	2	1				
Clinopodium_nepeta	1894	H SCAP	OROF S-EUROP	5	7	5	3	9	3	x	x	x	x
Condrilla_juncea	1894	H SCAP	S-EUROP-SUDSIB	8	7	5	3	8	X	x	x	x	x
Convonvulus_arvensis	1894	G RHIZ	PALEOTEMP	7	7	5	4	5	5				
Coronilla_scorpioides	1894	T SCAP	EURIMEDIT	11	9	5	2	7	2				
Crepis_setosa	1894	T SCAP	E-EURIMEDIT	11	9	6	2	8	2				
Cruciata_glabra	1894	H SCAP	EURASIAT	5	6	5	5	6	6		x		
Cucumis_melo	1894	H SCAND	COLTIV	10	8	X	5	5	7				
Cymbalaria_muralis	1894	T SCAP	N-EURIMEDIT	7	7	5	2	5	3	x	x	x	x
Cynodon_dactylon	1894	G RHIZ	COSMOPOL	8	8	5	4	X	4	x			
Daucus_carota	1894	H BIENNE	PALEOTEMP	8	6	5	4	5	4				
Digitaria_sanguinalis	1894	T SCAP	COSMOPOL	7	7	5	3	6	4				
Diplotaxis_muralis	1894	T SCAP	EURIMEDIT-SUBATL	8	8	3	3	5	5				x
Dysphania_botrys	1894	T SCAP	EURASIAT	8	9	5	3	X	6				

Dysphania_multifida	1894	H SCAP	AVV NATURALIZZ	8	7	5	2	5	5				
Echinochloa_crus-galli	1894	T SCAP	SUBCOSMOP	6	7	5	7	X	8				
Echium_vulgare	1894	H BIENNE	EUROP	9	7	5	4	5	4				
Eragrostis_cilianensis	1894	T SCAP	COSMOPOL	8	8	5	3	6	3				
Eragrostis_pilosa	1894	T SCAP	COSMOPOL	8	8	5	3	6	2				
Erigeron_canadensis	1894	T SCAP	AVV NATURALIZZ	8	6	5	5	X	7	x	x	x	x
Eriobotrya_japonica	1894	P SCAP	COLTIV	9	9	5	5	5	5	x	x		
Erophila_verna	1894	T SCAP	CIRCUMBOR	9	7	4	2	4	1				x
Erucastrum_gallicum	1894	H SCAP	W-EUROP (ATL)	8	7	3	3	7	2				
Euphorbia_maculata	1894	T SCAP	AVV NATURALIZZ	7	8	5	2	5	4				
Euphorbia_peplus	1894	T REPT	EURIMEDIT	11	7	2	1	X	1				
Ficus_carica	1894	P SCAP	EURIMEDIT-TURAN	7	8	6	X	5	X	x	x	x	x
Filago_germanica	1894	T SCAP	PALEOTEMP	8	7	5	3	4	2				
Fragraria_vesca	1894	CH REPT	EUROSIB	6	X	4	4	X	5	x			
Galium_aparine	1894	T SCAP	EURASIAT	6	X	5	4	5	5	x		x	x
Galium_lucidum	1894	H SCAP	EURIMEDIT	8	8	5	3	X	2				
Geranium_dissectum	1894	T SCAP	EURASIAT	7	8	5	2	5	2				
Geranium_molle	1894	T SCAP	EURASIAT	7	6	5	3	5	4				
Geranium_pusillum	1894	T SCAP	EURASIAT	7	7	5	4	5	6				
Heliotropium_amplexicaule	1894	CH FRUT	AVV NATURALIZZ	9	9	5	3	5	2				x
Herniaria_hirsuta	1894	T SCAP	PALEOTEMP	9	6	5	4	2	2				
Hordeum_murinum	1894	T SCAP	CIRCUMBOR	8	8	4	5	5	3	x	x	x	x
Hypochaeris_radicata	1894	H ROS	EUROP-CAUCAS	9	8	4	2	X	1	x	x	x	x
Ipomoea_indica	1894	G RHIZ	COLTIV	7	7	5	5	5	5	x			
Lactuca_sativa	1894	H BIENNE	COLTIV	11	5	5	6	4	5	x	x	x	x
Lactuca_serriola	1894	H BIENNE	S-EUROP-SUDSIB	9	7	7	4	6	4	x	x	x	x
Lamium_amplexicaule	1894	T SCAP	PALEOTEMP	7	7	5	4	5	7				
Lamium_purpureum	1894	T SCAP	EURASIAT	7	7	5	4	5	5				
Lapsana_cummunis	1894	T SCAP	PALEOTEMP	5	X	5	5	X	7				
Legousia_speculum-veneris	1894	T SCAP	EURIMEDIT	7	7	5	4	8	3	x			
Leontodon_hirtus	1894	H ROS	NW-STENOMEDIT	8	8	5	3	7	2				
Lepidium_coronopus	1894	T REPT	EURIMEDIT	8	8	5	3	4	2				
Lepidium_ruderale	1894	T SCAP	S-EUROP-SUDSIB	8	7	6	4	5	3				

Leucanthemum_vulgare	1894	H SCAP	EUROSIB	7	X	4	4	X	3	x	x		
Linaria_vulgaris	1894	H SCAP	EURASIAT	8	5	5	3	7	3				
Lolium _multiflorum	1894	T SCAP	EURIMEDIT	7	7	5	4	X	6				
Lolium _perenne	1894	H CAESP	CIRCUMBOR	8	5	4	5	X	7	x	x	x	x
Lotus_corniculatus	1894	H SCAP	PALEOTEMP	7	X	5	4	7	2				
Lotus_tenuis	1894	H SCAP	PALEOTEMP	9	7	5	6	7	7				
Lycopus_europaeus	1894	H SCAP	PALEOTEMP	7	6	5	9	X	7				
Malva_alcea	1894	H SCAP	CENTRO-EUROP	8	6	4	5	8	8	x			
Matricaria_chamomilla	1894	T SCAP	SUBCOSMOP	7	5	5	6	5	5	x	x	x	x
Matricaria_suaveolens	1894	T SCAP	AVV NATURALIZZ	8	5	5	5	7	8				
Medicago_lupulina	1894	T SCAP	PALEOTEMP	7	5	X	4	8	7				
Medicago_sativa	1894	H SCAP	EURASIAT	8	5	7	3	9	3	x	x	x	x
Melissa_officinalis	1894	H SCAP	EURIMEDIT	6	7	5	4	6	4	x			
Mentha_x piperita	1894	H SCAP	EURIMEDIT	7	6	5	8	8	6	x	x		
Mercurialis_annua	1894	T SCAP	PALEOTEMP	7	7	5	4	7	8				
Myosotis_arvensis	1894	T SCAP	EUROP-CAUCAS	6	5	5	5	X	6				
Onobrychis_viciifolia	1894	H SCAP	MEDIT-MONT	8	7	6	3	8	3				
Orlaya_grandiflora	1894	T SCAP	S-EUROP-SUDSIB	7	6	6	3	7	6				
Oxalis_corniculata	1894	CH REPT	EURIMEDIT	7	7	0	4	X	6	x	x	x	x
Panicum_miliaceum	1894	T SCAP	AVV NATURALIZZ	6	7	5	4	7	3				
Pantago_coronopus	1894	T SCAP	EURIMEDIT	8	7	5	7	7	4				
Pantago_lanceolata	1894	H ROS	EURASIAT	6	7	5	X	X	X	x	x	x	x
Papaver _rhoeas	1894	T SCAP	E-MEDIT-MONT	6	6	5	5	7	X	x			
Parietaria_judaica	1894	H SCAP	EURIMEDIT-MACARON	7	8	5	3	X	6	x	x	x	x
Parietaria_officinalis	1894	H SCAP	EUROP-CAUCAS	4	8	4	5	7	7	x	x	x	x
Paulownia_tomentosa	1894	P CAESP	AVV NATURALIZZ	6	7	5	5	5	2				
Persicaria_lapathifolia	1894	T SCAP	PALEOTEMP	6	6	5	7	X	8				
Phaseolus_vulgaris	1894	T SCAP	COLTIV	10	8	X	5	5	7				
Phleum_pratense	1894	H CAESP	CIRCUMBOR	7	6	5	5	6	6				
Phyla_nodiflora	1894	CH REPT	PANTROP	6	8	3	6	5	5				
Phytolacca_americana	1894	G RHIZ	AVV NATURALIZZ	9	8	5	5	5	4			x	x
Picris_hieracioides	1894	H SCAP	EUROSIB	8	X	5	4	8	4				
Plantago_major	1894	H ROS	EURASIAT	8	X	X	5	X	7	x	x	x	x

Plantago_media	1894	H ROS	EURASIAT	7	X	7	4	8	3				
Poa_annua	1894	T CAESP	COSMOPOL	7	X	5	6	X	8	x	x	x	x
Poligonum_aviculare	1894	T REPT	COSMOPOL	7	7	5	3	6	1	x	x	x	x
Poligonum_convolvulus	1894	T SCAP	CIRCUMBOR	8	7	4	4	5	3				
Polycarpon_tetraphyllum	1894	T SCAP	EURIMEDIT	7	7	5	4	5	6	x	x	x	x
Portulaca_oleracea	1894	T SCAP	SUBCOSMOP	7	8	5	4	7	7	x	x	x	x
Potentilla_argentea	1894	H SCAP	CIRCUMBOR	9	4	4	2	3	1	x			
Potentilla_reptans	1894	H ROS	PALEOTEMP	6	6	5	6	7	5	x			
Prunus_avium	1894	P SCAP	PONTICA	4	5	6	5	7	5	x	x	x	x
Prunus_cerasus	1894	P SCAP	PONTICA	9	7	6	5	5	5	x	x	x	x
Ranunculus _bulbosus	1894	H SCAP	NE-MEDIT-MONT	7	7	4	4	5	5	x			
Ranunculus _lanuginosum	1894	H SCAP	EUROP-CAUCAS	3	X	4	6	7	7				
Ranunculus _velutinus	1894	H SCAP	N-EURIMEDIT	6	8	5	5	6	5				
Reseda _odorata	1894	T SCAP	AVV NATURALIZZ	7	6	5	3	8	4				
Rhamnus_alaternus	1894	P CAESP	EURIMEDIT	4	9	5	2	4	4				
Rubus_ulmifolius	1894	NP	EURIMEDIT	5	8	5	4	5	8				
Rumex_pulcher	1894	H SCAP	EURIMEDIT	8	8	5	2	6	9				
Sagina_procumbens	1894	H CAESP	SUBCOSMOP	6	X	5	6	7	6	x			
Salvia_pratensis	1894	H SCAP	EURIMEDIT	8	6	6	4	8	4				
Salvia_verbenaca	1894	H SCAP	STENOMEDIT-ATL	8	8	4	3	5	7				
Samolus_valerandi	1894	H CAESP	COSMOPOL	7	6	4	8	X	6				
Sanguisorba_minor	1894	H SCAP	PALEOTEMP	7	6	5	3	8	2				
Satureja_hortensis	1894	T SCAP	EURIMEDIT	7	7	5	4	5	4				
Scleranthus_annuus	1894	T SCAP	PALEOTEMP	6	5	5	X	2	4	x	x	x	x
Sclerochloa_dura	1894	T SCAP	EURIMEDIT	8	8	5	2	5	2		x		
Sempervivum_tectorum	1894	CH SUCC	OROF S-EUROP	8	5	5	2	4	X		x		
Senecio_vulgaris	1894	T SCAP	EURIMEDIT	7	X	X	5	X	8	x	x	x	x
Setaria_viridis	1894	T SCAP	SUBCOSMOP	7	6	X	4	X	7				
Sinapis_alba	1894	T SCAP	E-MEDIT-MONT	8	10	5	3	7	2				
Sisymbrium_officinale	1894	T SCAP	PALEOTEMP	8	6	5	4	X	7				
Solanum_lycopersicum	1894	T SCAP	AVV NATURALIZZ	7	7	X	5	5	7	x		x	
Sonchus_oleraceus	1894	T SCAP	EURASIAT	7	5	X	4	8	8				
Stellaria_alsine	1894	H SCAP	CIRCUMBOR	5	4	4	8	4	4				

Stellaria_media	1894	T REPT	COSMOPOL	6	X	X	4	7	8	x	x	x	x
Tanacetum_parthenium	1894	H SCAP	E-EURIMEDIT	6	5	6	5	5	6				
Taraxacum_officinale	1894	H ROS	CIRCUMBOR	7	X	X	5	X	7	x	x	x	x
Thymus_serpyllum	1894	CH FRUT	W-STENOMEDIT	8	8	4	2	7	1				x
Trifolium _arvense	1894	T SCAP	PALEOTEMP	8	5	5	2	2	1				
Trifolium_dubium	1894	T SCAP	EUROP-CAUCAS	6	6	4	5	5	4				
Trifolium_nigrescens	1894	T SCAP	EURIMEDIT	8	6	5	5	5	6				
Trifolium_pratense	1894	CH PULV	EUROSIB	7	X	4	X	X	X	x	x	x	x
Trifolium_repens	1894	CH REPT	PALEOTEMP	8	X	X	X	X	7				
Trisetaria_flavescens	1894	H CAESP	EURASIAT	7	X	5	X	X	5				
Tussilago_farfara	1894	G RHIZ	PALEOTEMP	8	X	5	6	8	7	x			x
Urtica_dioica	1894	H SCAP	SUBCOSMOP	X	X	X	6	X	8	x	x	x	x
Valerianella_carinata	1894	T SCAP	EURIMEDIT	7	8	5	4	8	X				
Valerianella_dentata	1894	T SCAP	SUBATLANT	7	5	4	4	7	X				
Verbena_officinalis	1894	H SCAP	PALEOTEMP	9	5	5	4	X	6				
Veronica_arvensis	1894	T SCAP	PALEOTEMP	5	5	5	5	6	X				
Veronica_hederifolia	1894	T SCAP	EURASIAT	6	6	5	5	3	7				
Veronica_persica	1894	T SCAP	AVV NATURALIZZ	8	7	5	5	5	6				
Veronica_polita	1894	T SCAP	PALEOTEMP	5	6	5	4	8	7				
Viola_odorata	1894	H ROS	EURIMEDIT	5	6	5	5	X	8				
Vitis_vinifera	1894	P LIAN	COLTIV	6	8	5	6	8	6	x			
Zea_mays	1894	T SCAP	COLTIV	8	8	5	7	5	9	x	x	x	x
